# Photoinduced Bisphosphination of Alkynes with Phosphorus Interelement Compounds and Its Application to Double-Bond Isomerization

**DOI:** 10.3390/molecules27041284

**Published:** 2022-02-14

**Authors:** Yuki Yamamoto, Ryo Tanaka, Shintaro Kodama, Akihiro Nomoto, Akiya Ogawa

**Affiliations:** Department of Applied Chemistry, Graduate School of Engineering, Osaka Prefecture University, Osaka 599-8531, Japan; syb02137@edu.osakafu-u.ac.jp (Y.Y.); sab02088@edu.osakafu-u.ac.jp (R.T.); skodama@chem.osakafu-u.ac.jp (S.K.); nomoto@chem.osakafu-u.ac.jp (A.N.)

**Keywords:** interelement compounds, photoinduced bisphosphination, radical reaction, stereoselective synthesis, double-bond isomerization

## Abstract

The addition of interelement compounds with heteroatom-heteroatom single bonds to carbon-carbon unsaturated bonds under light irradiation is believed to be an atomically efficient method to procure materials with carbon-heteroatom bonds. In this study, we achieved the photoinduced bisphosphination of alkynes using the phosphorus interelement compound, tetraphenyldiphosphine monosulfide (**1**), to stereoselectively obtain the corresponding (*E*)-*vic*-1,2-bisphosphinoalkenes, which are important transition-metal ligands. The bisphosphination reaction was performed by mixing **1** and various alkynes and then exposing the mixture to light irradiation. Optimization of the conditions for the bisphosphination reaction resulted in a wide substrate range and excellent *trans*-selectivity. Moreover, the completely regioselective introduction of pentavalent and trivalent phosphorus groups to the terminal and internal positions of the alkynes, respectively, was achieved. We also found that the novel double-bond isomerization reaction of the synthesized bisphosphinated products occurred with a catalytic amount of a base under mild conditions. Our method for the photoinduced bisphosphination of carbon-carbon unsaturated compounds may have strong implications for both organic synthesis and organometallic and catalyst chemistry.

## 1. Introduction

**Scheme 1:**
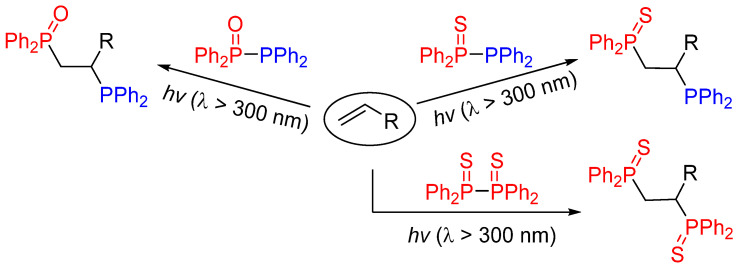
Photoinduced radical addition of phosphorus–phosphorus interelement compounds to alkenes.

The addition of interelement compounds with heteroatom-heteroatom single bonds to carbon-carbon unsaturated bonds has recently attracted wide attention as an atomically efficient method for carbon-heteroatom bond formation [1,2,3,4,5,6]. This addition reaction is promoted by transition-metal catalysts, acids, bases, and radical initiators [7,8,9,10,11,12,13,14,15,16,17,18,19,20,21,22,23,24,25,26]. On the other hand, photoirradiation has recently attracted much attention as a clean, eco-friendly, and powerful method in organic synthesis [27,28,29]. Thus, given the drawbacks of conventional methods, photoirradiation-induced radical addition reactions, which do not require additives, are becoming increasingly important from the viewpoint of green innovation [30,31]. The radical addition reactions of halogens and organic disulfides have been reported to occur under photoirradiation, but their synthetic applications are limited. To clarify the universality of the photoinduced radical reaction of heteroatom compounds as a method for generating carbon-heteroatom bonds, we previously investigated a series of radical addition reactions to the carbon-carbon unsaturated bonds, of group 16 (e.g., diselenides and ditellurides) [32,33,34,35,36], group 15 (e.g., diphosphines) [37,38,39,40,41,42], and group 13 (e.g., diboranes) compounds [43,44]. We then combined these reactions with disulfides and fluorinated iodoalkanes and successfully formed a variety of carbon-heteroatom bonds [45,46,47,48,49,50,51,52,53]. We also recently developed the radical addition reactions of diphosphines containing pentavalent phosphorus groups, such as Ph_2_P(O)-PPh_2_, Ph_2_P(S)-PPh_2_, and Ph_2_P(S)-P(S)Ph_2_, to alkenes (Scheme 1) [38,39,40]. Because the synthesized vicinally diphosphinated adducts are excellent ligands for transition metals [54,55,56,57,58], the development of a novel method for the photoinduced bisphosphination of carbon-carbon unsaturated compounds is expected to have a great impact on both organic synthesis and organometallic and catalyst chemistry.

As shown in Scheme 1, pentavalent and trivalent phosphorus groups can be simultaneously introduced to the terminal and internal positions of terminal alkenes, respectively, to obtain the corresponding adducts with excellent regioselectivity. This method can be used to obtain a variety of vicinal phosphines in a simple manner. The radical addition of diphosphines to alkynes generates *E*- and *Z*-isomers; therefore, the development of stereoselective methods to obtain *vic*-1,2-bisphosphinoalkenes may lead to the production of novel phosphorus ligands with the use of synthetic intermediates. However, only one example of the photoinduced radical addition of the diphosphine Ph_2_P(S)-PPh_2_ to alkynes has been studied thus far [39], and the details of the substrate scope, factors influencing the stereoselectivity of the adducts, and relevant synthetic applications have not been elucidated. 

**Scheme 2:**
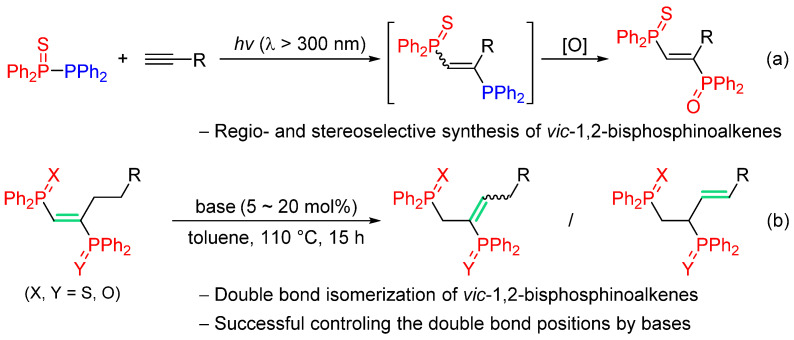
(**a**) Photoinduced bisphosphination of alkynes with phosphorus-based interelement compounds; (**b**) applications of *vic*-1,2-bisphosphinoalkenes to double-bond isomerization.

In this paper, we report the results of a detailed study on the radical addition reaction of Ph_2_P(S)-PPh_2_ to alkynes under light irradiation (Scheme 2a) and investigate the synthetic utilization of the generated *vic*-1,2-bisphosphinoalkenes. Interestingly, we found that the novel double-bond isomerization reaction of *vic*-1,2-bisphosphinoalkenes proceeded smoothly under mild conditions in the presence of a catalytic amount of a base (Scheme 2b). 

## 2. Results and Discussion

Obtaining a single isomer with the highest selectivity among several possible regio-and stereoisomers is necessary to develop a straightforward method for the synthesis of functional phosphorus-based ligands for metals. Therefore, we began our research by monitoring the time-dependent profiles of the photoinduced bisphosphination of alkynes with Ph_2_P(S)-PPh_2_ (**1**) by ^31^P NMR spectroscopy. The results are summarized in Table 1 and Table 2. When aliphatic 1-octyne **2a** was used as the substrate (Table 1), the *E*-isomer of **3a** was gradually formed with excellent stereoselectivity under light irradiation for up to 9 h; minimal formation of the *Z*-isomer was observed. After 9 h, the yield of *Z*-**3a** gradually increased and the stereoselectivity of *E*-**3a** decreased. 

Table 2 shows the results of the photoinduced addition reaction of **1** with phenylacetylene **2b** as an aromatic alkyne. The yield of *Z*-**3b** increased at a much shorter photoirradiation time with **2b** than with the aliphatic acetylene, thereby suggesting that photoirradiation led to the rapid isomerization of *E*-**3b** to *Z*-**3b**. These results indicate that the optimum reaction times for the selective synthesis of *E*-adducts are 9 h for aliphatic alkynes and 2 h for aromatic alkynes.

With the optimized conditions (aliphatic alkynes: 9 h, arylacetylenes: 2 h) in hand, we then evaluated the substrate scope of the photoinduced bisphosphination of a series of alkynes with **1** (Table 3). Because the formed *vic*-1,2-bisphosphinoalkene **3** has a trivalent phosphorus group in its structure and is, therefore, sensitive to air, it was successfully isolated by sequential oxidation to **4** at 25 °C for 30 min using 30% aqueous H_2_O_2_. As shown in Table 3, 1-octyne **2a** and phenylacetylene **2b** were successfully converted to the corresponding adducts **4a** and **4b**, respectively, in good yields with excellent stereoselectivity (*E*/*Z* = 90/10, entries 1 and 2). The phosphinylphosphination of alkynes under light irradiation could also be applied to various alkynes containing a branched chain (**2c**), cyclohexyl group (**2d**), benzyl group (**2e**), and phenethyl group (**2f**), and the corresponding adducts **4c**–**4f** were obtained in moderate yields with good stereoselectivity (*E*/*Z* = 89/11–100/0, entries 3–6). The use of ethyl propiolate **2h**, an electron-deficient alkyne, did not provide the desired adducts in sufficient yield, and a complex mixture was obtained after 9 h of irradiation (entry 7 in Table 3). The reaction was also applicable to an alkyne with a chloro group, and the desired adduct **4h** was obtained in 54% yield with excellent stereoselectivity (*E*/*Z* = 91/9, entry 8 in Table 3). Moreover, arylacetylenes **2i** and **2j** were successfully converted to the corresponding adducts **4i** and **4j** in 65% and 64% yields, respectively, after irradiation for 2 h, and the *E*-adducts were isolated as nearly pure isomers (entries 9 and 10). When 4-octyne, one of the internal alkynes, was used as a substrate, the photoinduced bisphosphination with **1** did not proceed at all, even after 9 h of irradiation, and **1** was recovered in 78% yield. This might be due to the steric hindrance of the substrate to Ph_2_P(S)-PPh_2_.

**Scheme 3:**
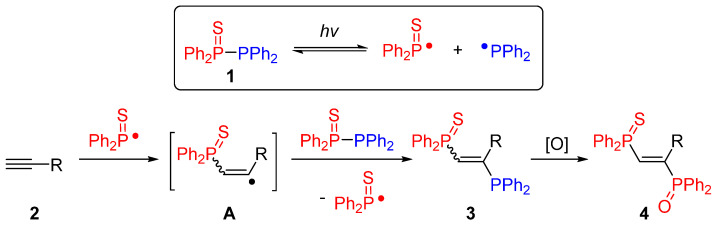
A plausible pathway for the photoinduced bisphosphination of alkyne with **1**.

Based on the results of this study and our previous studies, a plausible reaction pathway for the photoinduced bisphosphination of alkynes with **1** is shown in Scheme 3. In the initiation stage, homolytic cleavage of the P–P bond occurred reversibly under light to form Ph_2_P(S)• and Ph_2_P•. The generated Ph_2_P(S)• selectively attacks the terminal position of alkyne to form **A**. Then, the carbon radical **A** selectively reacts with the Ph_2_P-moiety of **1**, which is sterically less hindered than the Ph_2_P(S)-moiety. Finally, the following oxidation of the trivalent phosphorus group of the product **3** resulted in the corresponding *vic*-1,2-bisphosphinoalkene **4**.

**Scheme 4:**
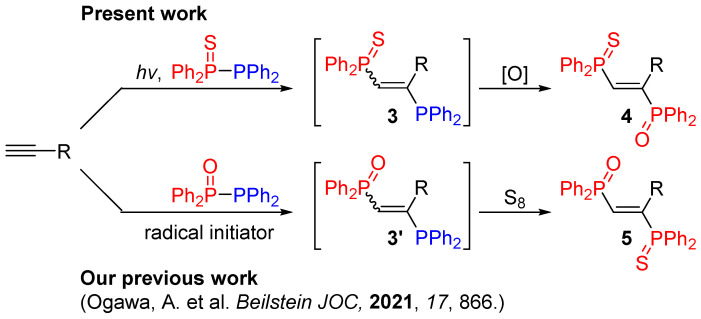
Regio-complementary synthesis of *vic*-1,2-bisphosphinoalkenes.

Our previous studies also showed that the addition reaction of Ph_2_P(O)–PPh_2_ to terminal alkynes can selectively introduce Ph_2_P(O) and Ph_2_P groups to the terminal and internal positions of alkynes, respectively, under light irradiation or in the presence of a catalytic radical initiator (e.g., AIBN, V-40, etc.). Treatment of adduct **3′** with elemental sulfur resulted in the formation of adduct **5** featuring Ph_2_P(O) and Ph_2_P(S) groups at the terminal and internal positions, respectively (Scheme 4) [38,59]. As shown in Table 3, a variety of *vic*-1,2-bisphosphinoalkenes **4** were obtained in good yields with remarkable stereoselectivity upon the addition of Ph_2_P(S)-PPh_2_ to the corresponding alkynes under light irradiation. Interestingly, our method allowed the regio-complementary introduction of Ph_2_P(S) and Ph_2_P(O) groups to the terminal and internal positions of alkynes, respectively, thereby providing a versatile synthetic approach to obtain bisphosphinated materials. The synthesized bisphosphinated products **4** and **5** could easily be reduced to afford the corresponding trivalent phosphine compounds, (*E*)-Ph_2_PCH=CR(PPh_2_), as monodentate ligands for mononuclear complexes. This feature is highly attractive because a hierarchical structure can be constructed by cross-linking the two metals [60,61,62,63].

In addition, if the position of the carbon-carbon double bond in the series of addition products **4** and **5** can be controlled by an isomerization reaction, it will be possible to prepare a more diverse group of phosphorus ligands. Therefore, we started to investigate the isomerization of the carbon-carbon double bond using various inorganic and organic bases. Here, regarding the synthesis and purification of **4** and **5**, the separation of **4** and Ph_2_P(S)-PPh_2_ was rather difficult, and **4** could only be purified on a small scale. In contrast, **5** could be synthesized on a gram scale and was easily isolated by silica gel column chromatography. Therefore, **5** was chosen as a model substrate for the isomerization reaction.

**Scheme 5:**
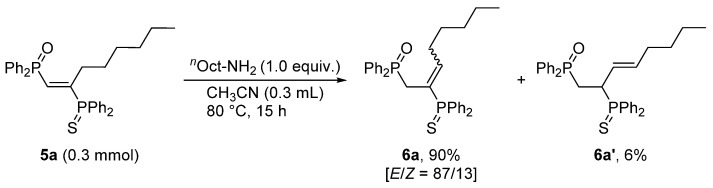
Base-promoted double-bond isomerization of *vic*-1,2-bisphosphinoalkene **5a**.

Interestingly, the isomerization of the carbon-carbon double bond of adduct **5** occurred when amines, which are representative organic bases, were used (Scheme 5). When adduct **5a** bearing an alkyl chain was treated with an equimolar amount of *n*-octylamine in acetonitrile at 80 °C for 15 h, the double-bond isomerization products **6a** and **6a’** were obtained in 90% and 6% yields, respectively.

To clarify the influence of the base, solvent, and temperature on the regioselectivity of the double-bond isomerization of **5a**, we performed detailed optimization studies of the reaction conditions, as shown in Table 4. When the amount of the base was reduced to 20 mol% and the isomerization reaction was attempted under toluene reflux conditions (110 °C), **6a** was obtained in high yield (90%, entry 2 in Table 4). This result indicates that the double-bond isomerization reaction proceeds with a catalytic amount of the base. When the amount of the base was reduced to 5 mol% and the reaction was performed under the same conditions, the yield of **6a** decreased (entry 3 in Table 4). When the base was changed from *n*-octylamine to 1,8-diazabicyclo[5.4.0]undec-7-ene (DBU), the yield of **6a** increased to 59%; very interestingly, **6a’** in 39% yield was also obtained (entry 4 in Table 4). When the amount of DBU was increased to 40 mol%, **6a’** was preferentially formed in 83% yield (entry 5 in Table 4). In the absence of the base, the isomerization did not occur (entry 6 in Table 4).

Next, we investigated the same double-bond isomerization reaction using the addition product **5b** with a phenethyl group (Table 5). Surprisingly, the isomerization reaction of **5b** using primary amines, such as *n*-butylamine and *n*-octylamine, in acetonitrile selectively produced **6b** as the sole product, which was formed by the double isomerization of the carbon-carbon double bond of **5b** (entries 1 and 2 in Table 5). This might be attributed to the C–C double bond of **6b** being stabilized by conjugation with aromatic rings. The use of secondary or tertiary amines resulted in the formation of very small amounts of **6b**, most likely because of the steric hindrance of the bases used (entries 3–5 in Table 5). When DBU was used as the base, **6b** was successfully obtained in 94% yield (entry 6 in Table 5). Other bases, such as 4-dimethylaminopyridine (DMAP) and Cs_2_CO_3_, were ineffective for double-bond isomerization (entries 7 and 8 in Table 5). Base-catalyzed isomerization was attempted using *n*-octylamine and DBU (20 mol%) in toluene at 110 °C, and **6b** was successfully obtained in excellent yield (entries 9 and 10 in Table 5). Moreover, the use of only 5 mol% DBU led to the nearly quantitative formation of **6b** (entry 11 in Table 5).

**Figure 1:**
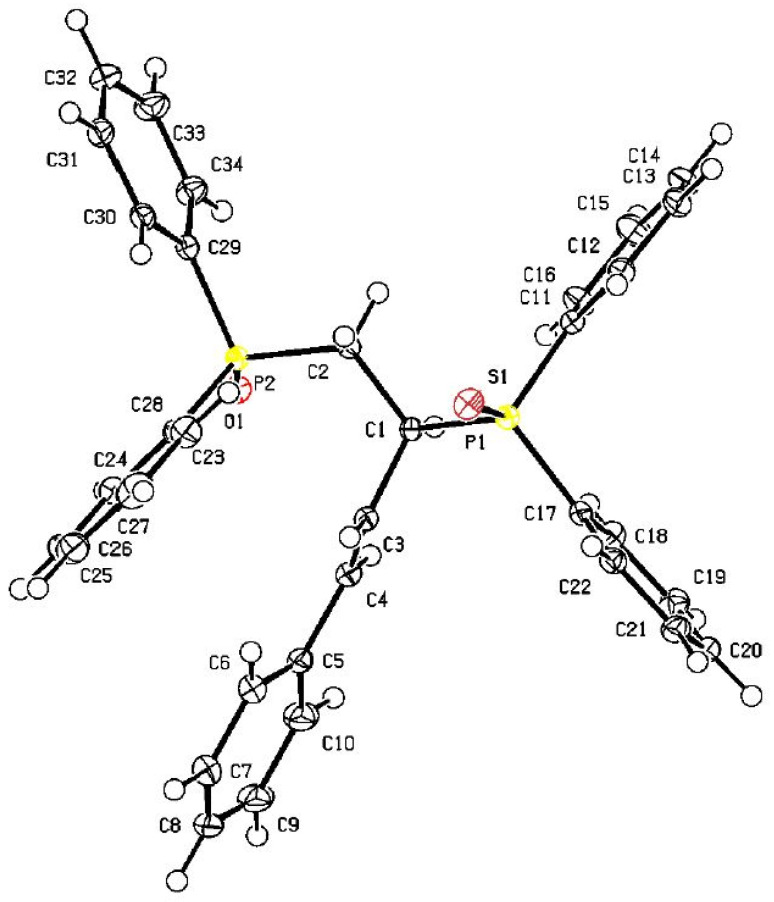
Crystal structure of **6b** with numbered atoms. Ellipsoids are shown at the 50% probability level. Selected interatomic distances (Å) and angles (deg): P1–S1, 1.9577(4); P1–C11, 1.8171(12); P1–C17, 1.8123(12); P1–C1, 1.8420(12); P2–O1, 1.4905(9); C2–C1, 1.5494(15); P2–C29, 1.8130(12); P2–C23, 1.8035(12); P2–C2, 1.8084(12); C4–C3, 1.3326(17); C3–C1, 1.5066(16); C5–C4, 1.4727(17); C11–P1–S1, 113.27(4); C11–P1–C1, 106.17(5); C17–P1–S1, 112.79(4); C17–P1–C11, 104.27(5); C17–P1–C1, 107.85(5); C1–C2–P2, 114.40(8); C1–P1–S1, 111.95(4); O1–P2–C29, 111.02(5); O1–P2–C23, 112.90(5); O1–P2–C2, 113.53(5); C23–P2–C29, 106.72(5); C23–P2–C2, 107.09(6); C2–P2–C29, 105.04(5); C30–C29–P2, 123.46(9); C3–C4–C5, 127.10(12); C34–C29–P2, 117.36(9); C4–C3–C1, 121.29(11); C12–C11–P1, 118.14(9); C2–C1–P1, 106.85(7); C3–C1–P1, 110.00(8); C16–C11–P1, 122.12(9); C3–C1–C2, 114.34(9); C24–C23–P2, 117.07(9); C9–C10–C5, 120.76(15); C28–C23–P2, 123.42(10); C22–C17–P1, 119.52(9); C18–C17–P1, 120.30(9); C6–C5–C4, 122.56(12); C10–C5–C4, 118.81(13).

Figure 1 represented the result of X-ray single-crystal structure analysis of the double-bond isomerization product **6b**. The result shown in Figure 1 clarifies the regio- and stereoselective formation of *E*-isomer as a single product, having different types of phosphorus functional groups in one molecule. Such a compound with both two phosphorus functional groups and one vinyl functional group is very rare; thus, the developed method in this work will be a powerful protocol for the facile preparation of a variety of new phosphorus ligands.

**Scheme 6:**
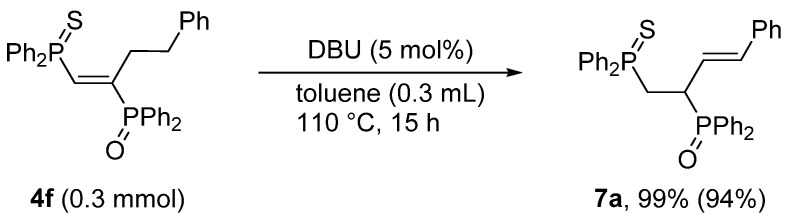
Base-catalyzed double-bond isomerization of **4f**.

We also performed the base-catalyzed isomerization reaction on the regio-complementary bisphosphination product **4f**, and successfully obtained **7a** in 99% yield with good regioselectivity (Scheme 6).

## 3. Materials and Methods

### 3.1. General Information

Unless otherwise stated, all starting materials were purchased from commercial sources and used without further purification. The diphosphine **1** was prepared according to the previously reported procedure [39]. All solvents were distilled and degassed with argon before use. ^1^H, ^13^C{^1^H}, and ^31^P NMR spectra were recorded in CDCl_3_ using a Bruker BioSpin Ascend 400 spectrometer (Tokyo, Japan) at 400, 100, and 162 MHz, respectively, with Me_4_Si as the internal standard. The characterization data of compounds are shown as follows (^1^H, ^13^C{^1^H}, and ^31^P NMR spectra are included in the Supplementary Materials).

### 3.2. General Procedure for the Photoinduced Bisphosphination of Alkynes with Tetraphenyldiphosphine Monosulfide

The diphosphine **1** (0.4 mmol), alkyne **2** (0.4 mmol), and degassed dry CH_2_Cl_2_ (0.4 mL) were added to a sealed Pyrex NMR tube under an argon atmosphere. The mixture was irradiated with a xenon lamp (100 W) at a distance of 10 cm for 2–9 h at 20–25 °C. Since **1** is sensitive to the reaction temperature [39], the NMR tube was immersed in water during light exposure to maintain a reaction temperature of 20–25 °C. After the reaction, the mixture was transferred to a 10 mL test tube with a stir bar and added with 30% H_2_O_2_ (0.4 mmol). The mixture was stirred at 25 °C for 30 min in air and quenched with saturated aqueous Na_2_S_2_O_3_ (3 mL). The resulting solution was extracted using CH_2_Cl_2_ (10 mL × 3). The organic layer was washed with saturated aqueous Na_2_S_2_O_3_ (10 mL) and brine (10 mL) and then dried with anhydrous MgSO_4_. The solvent was concentrated under reduced pressure. Finally, the residue was purified by silica gel column (AcOMe/iso-hexane) and preparative thin-layer (AcOMe/*iso*-hexane) chromatography to yield **4** (Table 3).

(*E*)-(1-(Diphenylphosphorothioyl)oct-1-en-2-yl)diphenylphosphine oxide (**4a**) (CAS: 2271208-25-2) [39]. After purification, the molar ratio of *E*-isomer/*Z*-isomer was 90/10. Colorless oil, 133.1 mg, 63%; ^1^H NMR (400 MHz, CDCl_3_): δ 7.84–7.79 (dd, *J* = 13.4, 7.0 Hz, 4H), 7.73–7.69 (dd, *J* = 11.6, 7.2 Hz, 4H), 7.57–7.54 (m, 2H), 7.50–7.44 (m, 6H), 7.43–7.38 (m, 4H), 7.20 (dd, *J*_H–P_ = 23.7, 21.6 Hz, 1H), 2.67–2.59 (m, 2H), 1.02–0.90 (m, 4H), 0.85–0.78 (m, 4H), 0.71 (t, *J* = 7.3 Hz, 3H); ^13^C{^1^H} NMR (100 MHz, CDCl_3_): δ 156.9 (d, *J*_C–P_ = 79.2 Hz), 136.2 (dd, *J*_C–P_ = 72.3, 8.1 Hz), 133.6 (d, *J*_C–P_ = 84.3 Hz), 132.4 (d, *J*_C–P_ = 2.6 Hz), 132.1 (d, *J*_C–P_ = 9.6 Hz), 131.7 (d, *J*_C–P_ = 2.9 Hz), 131.1 (d, *J*_C–P_ = 10.7 Hz), 130.9 (d, *J*_C–P_ = 100.9 Hz), 128.7 (d, *J*_C–P_ = 11.8 Hz), 128.6 (d, *J*_C–P_ = 12.4 Hz), 31.0, 30.6 (dd, *J*_C–P_ = 8.8, 8.8 Hz), 29.4, 28.9, 22.3, 14.0; ^31^P NMR (162 MHz, CDCl_3_): δ 30.3 (d, *J*_P–P_ = 56.0 Hz), 27.8 (d, *J*_P–P_ = 56.1 Hz) (for *Z*-isomer: δ 34.9 (d, *J*_P–P_ = 18.0 Hz), 26.0 (d, *J*_P–P_ = 18.0 Hz)).

(*E*)-2-((Diphenylphosphorothioyl)-1-phenylvinyl)diphenylphosphine oxide (**4b**). After purification, the molar ratio of *E*-isomer/*Z*-isomer was 90/10. White solid, 128.8 mg, 62%, mp 47.0–47.5 °C; ^1^H NMR (400 MHz, CDCl_3_): δ 7.69 (m, 9H), 7.51–7.47 (m, 2H), 7.41–7.37 (m, 4H), 7.32–7.28 (m, 2H), 7.25–7.20 (m, 4H), 6.93–6.86 (m, 3H), 6.81–6.78 (m, 2H); ^13^C {^1^H} NMR (100 MHz, CDCl_3_): δ 152.8 (d, *J*_C–P_ = 79.8 Hz), 137.0 (dd, *J*_C–P_ = 71.9, 7.9 Hz), 132.6, 132.3 (d, *J*_C–P_ = 9.2 Hz), 132.3 (d, *J*_C–P_ = 2.4 Hz), 132.1 (d, *J*_C–P_ = 84.9 Hz), 131.3 (d, *J*_C–P_ = 10.5 Hz), 131.28 (d, *J*_C–P_ = 3.2 Hz), 129.9 (dd, *J*_C–P_ = 4.5, 1.7 Hz), 129.7 (d, *J*_C–P_ = 102.5 Hz), 128.5 (d, *J*_C–P_ = 11.9 Hz), 128.3 (d, *J*_C–P_ = 12.4 Hz), 128.1 (d, *J*_C–P_ = 1.8 Hz), 127.3; ^31^P NMR (162 MHz, CDCl_3_): δ 30.0 (d, *J*_P–P_ = 48.5 Hz), 27.8 (d, *J*_P–P_ = 48.8 Hz) (for *Z*-isomer: δ 33.7 (d, *J*_P–P_ = 12.3 Hz), 23.1 (d, *J*_P–P =_ 12.3 Hz)); HRMS (EI) *m*/*z* calcd for C_32_H_26_OP_2_S [M]^+^: 520.1180, found: 520.1173.

(*E*)-(1-(Diphenylphosphorothioyl)-5-methylhex-1-en-2-yl)diphenylphosphine oxide (**4c**). White solid, 98.0 mg, 48%, mp 174.0–174.5 °C; ^1^H NMR (400 MHz, CDCl_3_): δ 7.85–7.80 (m, 4H), 7.73–7.68 (m, 4H), 7.58–7.54 (m, 2H), 7.50–7.45 (m, 6H), 7.45–7.40 (m, 4H), 7.28 (dd, *J*_H–P_ = 25.7, 21.7 Hz, 1H), 2.68–2.59 (m, 2H), 1.06–0.98 (m, 1H), 0.80–0.75 (m, 2H), 0.46 (d, *J* = 6.6 Hz, 6H); ^13^C{^1^H} NMR (100 MHz, CDCl_3_): δ 157.2 (d, *J*_C–P_ = 78.6 Hz), 136.4 (dd, *J*_C–P_ = 72.4, 8.0 Hz), 133.7 (d, *J*_C–P_ = 84.0 Hz), 132.3 (d, *J*_C–P_ = 2.8 Hz), 132.2 (d, *J*_C–P_ = 9.6 Hz), 131.6 (d, *J*_C–P_ = 2.9 Hz), 131.2 (d, *J*_C–P_ = 10.5 Hz), 131.0 (d, *J*_C–P_ = 101.0 Hz), 128.7 (d, *J*_C–P_ = 11.9 Hz), 128.7 (d, *J*_C–P_ = 12.2 Hz), 37.1, 28.9 (dd, *J*_C–P_ = 8.9, 9.1 Hz), 28.4, 21.8; ^31^P NMR (162 MHz, CDCl_3_): δ 30.0 (d, *J*_P–P_ = 55.8 Hz), 27.6 (d, *J*_P–P_ = 55.1 Hz); HRMS (EI) *m*/*z* calcd for C_31_H_32_OP_2_S [M]^+^: 514.1649, found: 514.1644.

(*E*)-(1-Cyclohexyl-2-(diphenylphosphorothioyl)vinyl)diphenylphosphine oxide (**4d**). After purification, the molar ratio of *E*-isomer/*Z*-isomer was 89/11. Colorless oil, 89.2 mg, 42%; ^1^H NMR (400 MHz, CDCl_3_): δ 7.75–7.69 (m, 8H), 7.58–7.54 (m, 2H), 7.51–7.47 (m, 4H), 7.45–7.43 (m, 2H), 7.41–7.37 (m, 4H), 6.52 (dd, *J*_H–P_ = 24.3, 21.4 Hz, 1H), 3.23–3.11 (m, 1H), 1.80–1.70 (m, 2H), 1.46–1.37 (m, 3H), 1.26–1.22 (m, 2H), 1.11–1.01 (m, 1H), 0.75–0.65 (m, 2H); ^13^C{^1^H} NMR (100 MHz, CDCl_3_): δ 160.6 (d, *J*_C–P_ = 77.5 Hz), 136.7 (dd, *J*_C–P_ = 73.3, 10.2 Hz), 133.8 (d, *J*_C–P_ =83.3 Hz), 132.4 (d, *J*_C–P_ = 99.9 Hz), 132.1 (d, *J*_C–P_ = 2.7 Hz), 131.9 (d, *J*_C–P_ = 9.5 Hz), 131.6 (d, *J*_C–P_ = 3.0 Hz), 130.9 (d, *J*_C–P_ = 10.4 Hz), 128.6 (d, *J*_C–P_ = 12.2 Hz, *ortho* carbons of Ph_2_P(S) and Ph_2_P(O) group are overlapped), 44.5 (dd, *J*_C–P_ = 10.0, 7.2 Hz), 30.4, 26.1, 25.2; ^31^P NMR (162 MHz, CDCl_3_): δ 33.3 (d, *J*_P–P_ = 55.8 Hz), 27.5 (d, *J*_P–P_ = 54.3 Hz) (for *Z*-isomer: δ 35.0 (d, *J*_P–P_ = 18.2 Hz), 26.4 (d, *J*_P–P_ = 18.7 Hz)); HRMS (EI) *m*/*z* calcd for C_32_H_32_OP_2_S [M]^+^: 526.1649, found: 526.1649.

(*E*)-(1-(Diphenylphosphorothioyl)-3-phenylprop-1-en-2-yl)diphenylphosphine oxide (**4e**). After purification, the molar ratio of *E*-isomer/*Z*-isomer was 90/10. Colorless oil, 122.5 mg, 57%; ^1^H NMR (400 MHz, CDCl_3_): δ 7.78–7.73 (m, 4H), 7.57–7.52 (m, 4H), 7.44–7.40 (m, 4H), 7.38–7.30 (m, 8H), 7.25–7.16 (m, 1H), 7.21 (dd, *J*_H–P_ = 21.5, 21.2 Hz, 1H), 6.95–6.94 (m, 2H), 6.85–6.79 (m, 2H), 4.17 (d, *J*_H–P_ = 16.7 Hz, 2H); ^13^C{^1^H} NMR (100 MHz, CDCl_3_): δ 155.3 (d, *J*_C–P_ = 80.2 Hz), 137.6 (dd, *J*_C–P_ = 71.6, 8.1 Hz), 135.6, 133.4 (d, *J*_C–P_ = 84.2 Hz), 132.0 (d, *J*_C–P_ = 2.3 Hz), 131.9 (d, *J*_C–P_ = 9.7 Hz), 131.7 (d, *J*_C–P_ = 2.9 Hz), 131.0 (d, *J*_C–P_ = 10.5 Hz), 130.6 (d, *J*_C–P_ = 101.6 Hz), 129.7, 128.8 (d, *J*_C–P_ = 12.6 Hz), 128.4 (d, *J*_C–P_ = 12.1 Hz), 127.7, 126.1, 35.5 (dd, *J*_C–P_ = 9.2, 8.9 Hz); ^31^P NMR (162 MHz, CDCl_3_): δ 30.7 (d, *J*_P–P_ = 52.6 Hz), 27.9 (d, *J*_P–P_ = 52.3 Hz) (for *Z*-isomer: δ 33.4 (d, *J*_P–P_ = 16.5 Hz), 26.1 (d, *J*_P–P_ = 17.7 Hz)); HRMS (EI) *m*/*z* calcd for C_33_H_28_OP_2_S [M]^+^: 534.1336, found: 534.1337.

(*E*)-(1-(Diphenylphosphorothioyl)-4-phenylbut-1-en-2-yl)diphenylphosphine oxide (**4f**). After purification, the molar ratio of *E*-isomer/*Z*-isomer was 98/2. White solid, 109.8 mg, 50%, mp 179.5–180.0 °C; ^1^H NMR (400 MHz, CDCl_3_): δ 7.90–7.84 (m, 4H), 7.76–7.70 (m, 4H), 7.61–7.56 (m, 2H), 7.52–7.40 (m, 11H), 7.12–7.02 (m, 3H), 6.77–6.75 (m, 2H), 3.01–2.93 (m, 2H), 2.29–2.24 (m, 2H); ^13^C{^1^H} NMR (100 MHz, CDCl_3_): δ 156.0 (d, *J*_C–P_ = 77.9 Hz), 140.9, 137.0 (dd, *J*_C–P_ = 71.0, 7.6 Hz), 133.7 (d, *J*_C–P_ = 83.7 Hz), 132.6 (d, *J*_C–P_ = 2.7 Hz), 132.2 (d, *J*_C–P_ = 9.6 Hz), 131.8 (d, *J*_C–P_ = 2.8 Hz), 131.2 (d, *J*_C–P_ = 10.4 Hz), 130.8 (d, *J*_C–P_ = 105.6 Hz), 128.9 (d, *J*_C–P_ = 11.8 Hz), 128.8 (d, *J*_C–P_ = 12.3 Hz), 128.3, 128.2, 34.8, 32.8 (dd, *J*_C–P_ = 9.0, 8.8 Hz); ^31^P NMR (162 MHz, CDCl_3_): δ 29.4 (d, *J*_P–P_ = 54.2 Hz), 27.7 (d, *J*_P–P_ = 54.3 Hz) (for *Z*-isomer: δ 34.2 (d, *J*_P–P_ = 16.4 Hz), 25.8 (d, *J*_P–P_ = 16.9 Hz)); HRMS (EI) *m*/*z* calcd for C_34_H_30_OP_2_S [M]^+^: 548.1493, found: 548.1496.

(*E*)-(6-Chloro-1-(diphenylphosphorothioyl)hex-1-en-2-yl)diphenylphosphine oxide (**4h**). After purification, the molar ratio of *E*-isomer/*Z*-isomer was 91/9. White solid, 122.6 mg, 54%, mp 168.5–169.0 °C; ^1^H NMR (400 MHz, CDCl_3_): δ 7.83–7.77 (m, 4H), 7.73–7.68 (m, 4H), 7.60–7.56 (m, 2H), 7.52–7.47 (m, 6H), 7.45–7.40 (m, 4H), 7.20 (dd, *J*_H–P_ = 23.7, 21.3 Hz, 1H), 3.12 (t, *J* = 6.9 Hz, 2H), 2.72–2.64 (m, 2H), 1.37–1.30 (m, 2H), 1.17–1.09 (m, 2H); ^13^C{^1^H} NMR (100 MHz, CDCl_3_): δ 156.2 (d, *J*_C–P_ = 79.2 Hz), 136.8 (dd, *J*_C–P_ = 71.5, 8.0 Hz), 133.5 (d, *J*_C–P_ = 84.1 Hz), 132.5 (d, *J*_C–P_ = 2.7 Hz), 132.1 (d, *J*_C–P_ = 9.5 Hz), 131.8 (d, *J*_C–P_ = 2.8 Hz), 131.1 (d, *J*_C–P_ = 10.7 Hz), 130.6 (d, *J*_C–P_ = 101.4 Hz), 128.8 (d, *J*_C–P_ = 12.0 Hz), 128.8 (d, *J*_C–P_ = 12.3 Hz), 44.1, 32.7, 29.7 (dd, *J*_C–P_ = 9.0, 9.0 Hz), 26.5; ^31^P NMR (162 MHz, CDCl_3_): δ 30.3 (d, *J*_P–P_ = 54.3 Hz), 27.7 (d, *J*_P–P_ = 54.3 Hz) (for *Z*-isomer: δ 34.8 (d, *J*_P–P_ = 18.0 Hz), 25.9 (d, *J*_P–P_ = 17.0 Hz)); HRMS (EI) *m*/*z* calcd for C_30_H_29_ClOP_2_S [M]^+^: 534.1103, found: 534.1104.

(*E*)-(1-(4-(*tert*-Butyl)phenyl)-2-(diphenylphosphorothioyl)vinyl)diphenylphosphine oxide (**4i**). After purification, the molar ratio of *E*-isomer/*Z*-isomer was 99/1. White solid, 148.6 mg, 65%, mp 179.0–179.5 °C; ^1^H NMR (400 MHz, CDCl_3_): δ 7.67–7.61 (m, 8H), 7.50–7.45 (m, 3H), 7.42–7.37 (m, 4H), 7.28–7.24 (m, 2H), 7.21–7.17 (m, 4H), 6.90–6.88 (m, 2H), 6.81–6.79 (m, 2H), 1.12 (s, 9H); ^13^C{^1^H} NMR (100 MHz, CDCl_3_): δ 152.4 (d, *J*_C–P_ = 79.4 Hz), 150.9, 138.2 (dd, *J*_C–P_ = 72.2, 8.9 Hz), 132.3 (d, *J*_C–P_ = 9.4 Hz), 131.7 (d, *J*_C–P_ = 109.5 Hz), 131.3 (d, *J*_C–P_ = 10.7 Hz), 131.2, 131.0 (d, *J*_C–P_ = 96.8 Hz), 129.6 (dd, *J*_C–P_ = 4.4, 1.7 Hz), 129.7, 129.5, 128.5 (d, *J*_C–P_ = 12.1 Hz), 128.2 (d, *J*_C–P_ = 12.4 Hz), 124.3, 34.3, 31.1; ^31^P NMR (162 MHz, CDCl_3_): δ 30.3 (d, *J*_P–P_ = 50.1 Hz), 28.6 (d, *J*_P–P_ = 50.2 Hz) (for *Z*-isomer: δ 33.5 (d, *J*_P–P_ = 14.0 Hz), 23.3 (d, *J*_P–P_ = 12.1 Hz)); HRMS (EI) *m*/*z* calcd for C_36_H_34_OP_2_S [M]^+^: 576.1806, found: 576.1801.

(*E*)-(1-(4-Bromophenyl)-2-(diphenylphosphorothioyl)vinyl)diphenylphosphine oxide (**4j**). White solid, 152.3 mg, 64%, mp 186.7–187.3 °C; ^1^H NMR (400 MHz, CDCl_3_): δ 7.68–7.59 (m, 8H), 7.54–7.48 (m, 3H), 7.45–7.41 (m, 4H), 7.37–7.33 (m, 2H), 7.28–7.24 (m, 4H), 6.93–6.91 (m, 2H), 6.78–6.77 (m, 2H); ^13^C{^1^H} NMR (100 MHz, CDCl_3_): δ 151.5 (d, *J*_C–P_ = 79.6 Hz), 139.4 (dd, *J*_C–P_ = 71.4, 8.0 Hz), 132.5 (d, *J*_C–P_ = 2.5 Hz), 132.3 (d, *J*_C–P_ = 9.4 Hz), 131.9 (d, *J*_C–P_ = 54.5 Hz), 131.7, 131.5 (dd, *J*_C–P_ = 4.1, 2.0 Hz), 131.4 (d, *J*_C–P_ = 2.9 Hz), 131.3 (d, *J*_C–P_ = 10.6 Hz), 130.5, 129.4 (d, *J*_C–P_ = 102.7 Hz), 128.7 (d, *J*_C–P_ = 12.1 Hz), 128.4 (d. *J*_C–P_ = 12.6 Hz), 122.7 (d, *J*_C–P_ = 2.5 Hz); ^31^P NMR (162 MHz, CDCl_3_): δ 29.9 (d, *J*_P–P_ = 48.1 Hz), 27.9 (d, *J*_P–P_ = 48.1 Hz); HRMS (EI) calcd for C_32_H_25_BrOP_2_S [M]^+^: 598.0285, found: 598.0287.

### 3.3. Base-Catalyzed Double Bond Isomerization of vic-1,2-Bisphosphinoalkenes ***5a***

Degassed dry toluene (0.3 mL), **5a** (0.3 mmol), and 1-octylamine (20 mol%) were added to a 10 mL two-neck flask, and stirred for 15 h at 110 °C in an oil bath. The resulting solution was transferred to a round-bottom flask with acetone (5 mL), and the solvent was removed under reduced pressure. Finally, the residue was purified by preparative thin-layer chromatography (AcOMe/*iso*-hexane = 1:3) to give product **6a** (entry 2 in Table 4).

(*E*)-(2-(Diphenylphosphorothioyl)oct-2-en-1-yl)diphenylphosphine oxide (**6a**). After purification, the molar ratio of *E*-isomer/*Z*-isomer was 98/2. Colorless oil, 66.4 mg, 42%; ^1^H NMR (400 MHz, CDCl_3_): δ 7.75–7.70 (m, 4H), 7.57–7.52 (m, 4H), 7.39–7.24 (m, 12H), 6.00–5.90 (m, 1H), 3.96 (dd, *J*_H–P_ = 16.2, 13.5 Hz, 2H), 2.55–2.48 (m, 2H), 1.35–1.30 (m, 2H), 1.28–1.12 (m, 4H), 0.82 (t, *J* = 7.1 Hz, 3H); ^13^C{^1^H} NMR (100 MHz, CDCl_3_): δ 153.0 (dd, *J*_C–P_ = 7.6, 7.6 Hz), 133.4 (d, *J*_C–P_ = 98.1 Hz), 132.2 (d, *J*_C–P_ = 10.4 Hz), 131.7 (d, *J*_C–P_ = 84.5 Hz), 131.4 (d, *J*_C–P_ = 2.8 Hz), 131.3 (d, *J*_C–P_ = 3.0 Hz), 130.8 (d, *J*_C–P_ = 9.3 Hz), 128.29 (d, *J*_C–P_ = 11.4 Hz), 128.28 (d, *J*_C–P_ = 11.4 Hz), 122.9 (dd, *J*_C–P_ = 78.6, 8.8 Hz), 31.5 (d, *J*_C–P_ = 1.5 Hz), 31.4, 29.4 (dd, *J*_C–P_ = 66.0, 14.6 Hz), 28.1, 22.4, 14.0; ^31^P NMR (162 MHz, CDCl_3_): δ 50.9 (d, *J*_P–P_ = 9.5 Hz), 27.3 (d, *J*_P–P_ = 8.3 Hz) (for *Z*-isomer: δ 48.9 (d, *J*_P–P_ = 58.3 Hz), 19.6 (d, *J*_P–P_ = 58.6 Hz)); HRMS (EI) calcd for C_32_H_34_OP_2_S [M]^+^: 528.1806, found: 528.1807.

### 3.4. Base-Catalyzed Double Bond Isomerization of vic-1,2-Bisphosphinoalkenes ***5b***

Degassed dry toluene (1.2 mL), **5b** (1.2 mmol), and DBU (5 mol%) were added to a 10 mL two-neck flask, and stirred for 15 h at 110 °C in an oil bath. The resulting solution was transferred to a round-bottom flask with CH_2_Cl_2_ (5 mL), and the solvent was removed under reduced pressure. Finally, the residue was purified by recrystallization (*iso*-hexane/CH_2_Cl_2_) to give pure product **6b** (entry 11 in Table 5).

(*E*)-(2-(Diphenylphosphorothioyl)-4-phenylbut-3-en-1-yl)diphenylphosphine oxide (**6b**). White solid, 624.3 mg, 95%, mp 189.0–189.5 °C; ^1^H NMR (400 MHz, CDCl_3_): δ 8.14–8.09 (m, 2H), 7.78–7.73 (m, 2H), 7.69–7.64 (m, 2H), 7.61–7.56 (m, 2H), 7.53–7.51 (m, 3H), 7.44–7.40 (m, 1H), 7.37–7.31 (m, 3H), 7.30–7.25 (m, 2H), 7.22–7.15 (m, 3H), 7.10–7.03 (m, 3H), 6.69–6.67 (m, 2H), 5.88 (dd, *J*_H–H_ = 15.8 Hz, *J*_H–P_ = 5.1 Hz, 1H), 5.69–5.61 (m, 1H), 4.35–4.25 (m, 1H), 2.97–2.87 (m, 1H), 2.64–2.53 (m, 1H); ^13^C{^1^H} NMR (100 MHz, CDCl_3_): δ 136.2 (d, *J*_C–P_ = 3.6 Hz), 136.1 (d, *J*_C–P_ = 13.0 Hz), 134.0 (d, *J*_C–P_ = 98.9 Hz), 132.3 (d, *J*_C–P_ = 91.3 Hz), 132.0 (d, *J*_C–P_ = 2.8 Hz), 131.83 (d, *J*_C–P_ = 9.2 Hz), 131.78 (d, *J*_C–P_ = 8.7 Hz), 131.84 (d, overlapped), 131.5 (d, *J*_C–P_ = 2.8 Hz), 131.3 (d, *J*_C–P_ = 2.9 Hz), 131.1 (d, *J*_C–P_ = 9.5 Hz), 130.8 (d, *J*_C–P_ = 80.1 Hz), 130.4 (d, *J*_C–P_ = 75.0 Hz), 130.3 (d, *J*_C–P_ = 9.4 Hz), 129.1 (d, *J*_C–P_ = 11.6 Hz), 128.7 (d, *J*_C–P_ = 11.6 Hz), 128.4 (d, *J*_C–P_ = 11.9 Hz), 128.2 (d, *J*_C–P_ = 12.0 Hz), 127.9, 127.5, 126.3 (d, *J*_C–P_ = 1.9 Hz), 122.8 (dd, *J*_C–P_ = 7.5, 1.6 Hz), 38.8 (dd, *J*_C–P_ = 51.9, 2.8 Hz), 29.3 (d, *J*_C–P_ = 68.9 Hz); ^31^P NMR (162 MHz, CDCl_3_): δ 51.7 (d, *J*_P–P_ = 51.7 Hz), 29.8 (d, *J*_P–P_ = 51.2 Hz); HRMS (EI) *m*/*z* calcd for C_34_H_30_OP_2_S [M]^+^: 548.1493, found: 548.1502.

### 3.5. X-ray Diffraction Studies of ***6b***

An X-ray crystallographic measurement was carried out on a Rigaku VariMax RAPID diffractometer (Tokyo, Japan) with Mo-*K*α radiation at 103 K. Of 48,252 reflections collected, 6590 were unique (R*_int_* = 0.0233). Using Olex2, [64] the structure of **6b** was solved with the SHELXT [65] structure solution program using Intrinsic Phasing and refined with the SHELXL [66] refinement package using least squares minimization.

Crystallographic data: formula weight = 548.58; monoclinic; space group *P*2_1_/*c*; *a* = 11.2160(2) Å, *b* = 19.5271(4) Å, *c* = 13.8308(3) Å; *V* = 2874.78(15) Å^3^; *Z* = 4; *ρ*_calcd_ = 1.267 g cm^−3^; total reflections collected = 48252; *GOF* = 1.058; *R*_1_ = 0.0314; *wR*_2_ = 0.0810. Crystallographic data have been deposited with Cambridge Crystallographic Data Centre (CCDC-2132957). These data can be obtained free of charge via http://www.ccdc.cam.ac.uk/conts/retrieving.html (accessed on: 7 January 2022) (or from the CCDC, 12 Union Road, Cambridge CB2 1EZ, UK; Fax: +44 1223 336033; E-mail: deposit@ccdc.cam.ac.uk).

### 3.6. Base-Catalyzed Double Bond Isomerization of vic-1,2-Bisphosphinoalkenes ***4f***

Degassed dry toluene (0.3 mL), **4f** (0.3 mmol), and DBU (5 mol%) were added to a 10 mL two-neck flask, and stirred for 15 h at 110 °C in an oil bath. The resulting solution was transferred to a round-bottom flask with CH_2_Cl_2_ (5 mL), and the solvent was removed under reduced pressure. Finally, the residue was purified by recrystallization (*iso*-hexane/CH_2_Cl_2_) to give pure product **7a** (Scheme 5).

(*E*)-(1-(Diphenylphosphorothioyl)-4-phenylbut-3-en-2-yl)diphenylphosphine oxide (**7a**). White solid, 154.9 mg, 94%, mp 190.0–190.5 °C; ^1^H NMR (400 MHz, CDCl_3_): δ 8.01–7.99 (m, 2H), 7.79–7.74 (m, 2H), 7.70–7.64 (m, 4H), 7.54 (m, 3H), 7.40–7.32 (m, 6H), 7.13–7.05 (m, 6H), 6.64–6.63 (m, 2H), 5.96 (dd, *J*_H–H_ = 15.8 Hz, *J*_H–P_ = 4.0 Hz, 1H), 5.51–5.44 (m, 1H), 4.31–4.20 (m, 1H), 3.31–3.23 (m, 1H), 2.67–2.57 (m, 1H); ^13^C{^1^H} NMR (100 MHz, CDCl_3_): δ 136.5 (d, *J*_C–P_ = 11.2 Hz), 136.2 (d, *J*_C–P_ = 3.0 Hz), 134.3 (d, *J*_C–P_ = 82.7 Hz), 132.2 (d, *J*_C–P_ = 2.7 Hz), 131.74 (d, *J*_C–P_ = 10.5 Hz), 131.72 (d, *J*_C–P_ = 1.9 Hz), 131.5 (d, overlapped), 131.4 (d, *J*_C–P_ = 8.5 Hz), 131.38 (d, *J*_C–P_ = 99.8 Hz), 131.2 (d, *J*_C–P_ = 8.9 Hz), 131.0 (d, *J*_C–P_ = 2.7 Hz), 130.4 (d, *J*_C–P_ = 9.9 Hz), 131.0 (d, *J*_C–P_ = 87.5 Hz), 130.6 (d, *J*_C–P_ = 93.4 Hz), 129.1 (d, *J*_C–P_ = 11.4 Hz), 128.8 (d, *J*_C–P_ = 12.0 Hz), 128.3 (d, *J*_C–P_ = 11.7 Hz), 128.2 (d, *J*_C–P_ = 12.3 Hz), 127.9, 127.4, 126.1 (d, *J*_C–P_ = 1.0 Hz), 121.8 (dd, *J*_C–P_ = 9.3, 1.0 Hz), 39.1 (dd, *J*_C–P_ = 66.2, 2.3 Hz), 30.4 (d, *J*_C–P_ = 54.5 Hz); ^31^P NMR (162 MHz, CDCl_3_): δ 42.8 (d, *J*_P–P_ = 54.4 Hz), 35.7 (d, *J*_P–P_ = 54.4 Hz); HRMS (EI) *m*/*z* calcd for C_34_H_30_OP_2_S [M]^+^: 548.1493, found: 548.1502.

## 4. Conclusions

In this study, we achieved the photoinduced bisphosphination of alkynes using the phosphorous interelement compound Ph_2_P(S)-PPh_2_ to obtain the corresponding *vic*-1,2-bisphosphinoalkenes. Optimization of the reaction conditions resulted in a wide substrate range and excellent *trans*-selectivity. Moreover, the completely regioselective introduction of pentavalent and trivalent phosphorus groups to the terminal and internal positions of the alkynes, respectively, was achieved.

**Scheme 7:**
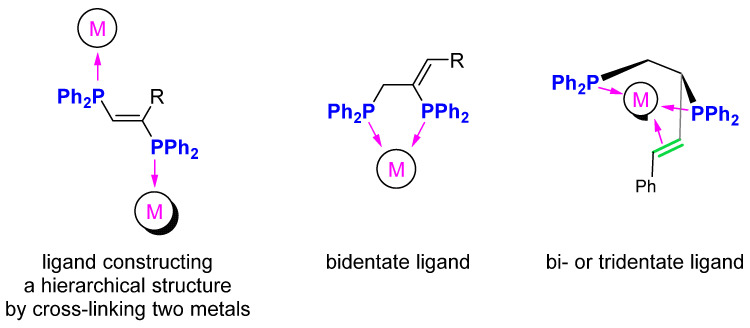
Possible metal ligands from *vic*-diphosphine compounds.

We found that the novel double-bond isomerization reaction of the synthesized 1,2-bisphosphinated products occurs with a catalytic amount of the base under mild conditions. Our method for the photoinduced bisphosphination of carbon-carbon unsaturated compounds may have strong implications for both organic synthesis and organometallic and catalyst chemistry. For example, several diphosphine compounds are believed to be useful as monodentate, bidentate, and tridentate ligands for various metals (Scheme 7).

## Figures and Tables

**Table 1:** Time-dependent profiles of the photoinduced bisphosphination of 1-octyne **2a**.

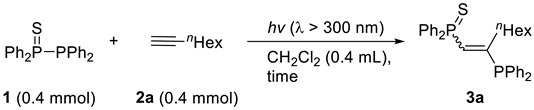				
**Time (h)**	**Yield 3a (%) ^a^**		***E*** **-Selectivity (%)**	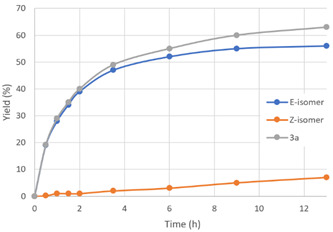
	***E***	***Z***		
0.5	19	0.2	99	
1.0	28	1	97	
1.5	34	1	97	
2.0	39	1	98	
3.5	47	2	96	
6.0	52	3	95	
**9.0**	**55**	**5**	**92**	
13	56	7	89	

^a^ Yields were determined by ^31^P NMR spectroscopy.

**Table 2:** Time-dependent profiles of the bisphosphination of phenylacetylene **2b**.

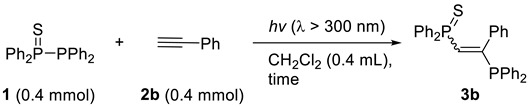				
**Time (h)**	**Yield 3b (%) ^a^**		***E*** **-Selectivity (%)**	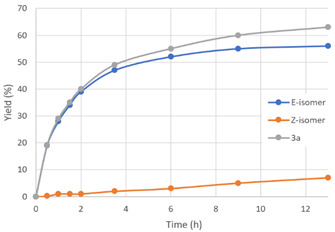
	***E***	***Z***		
0.5	41	2	95	
1.0	51	3	94	
**2.0**	**65**	**8**	**89**	
4.5	64	14	82	
6.0	61	19	76	
12	52	32	62	

^a^ Yields were determined by ^31^P NMR spectroscopy.

**Table 3:** Substrate scope for the photoinduced bisphosphination of alkynes with tetraphenyldiphosphine monosulfide (**1**).

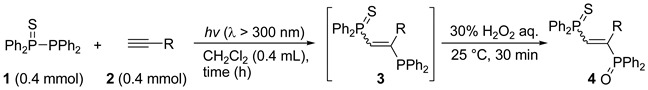					
**Entry**	**Alkyne 2**	**3**	**Yield (%) ^a^ [*E*/*Z*]**	**Product 4**	**Yield (%) ^b^ [*E*/*Z*]**
1 ^c^	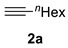	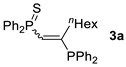	57 [91/9]	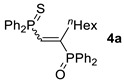	63 [90/10]
2 ^d^	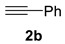	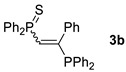	67 [90/10]	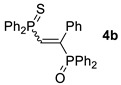	62 [90/10]
3 ^c^	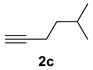	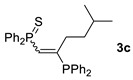	58 [91/9]	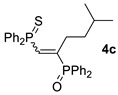	48 [100/0]
4 ^c^	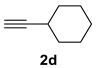	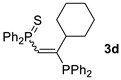	41 [90/10]	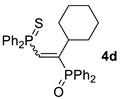	42 [89/11]
5 ^c^	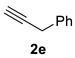	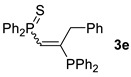	50 [90/10]	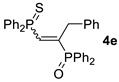	57 [90/10]
6 ^c^	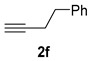	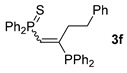	56 [89/11]	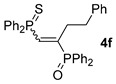	50 [98/2]
7 ^c^	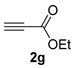	complex mixture	-	-	-
8 ^c^	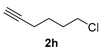	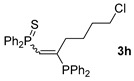	overlapped	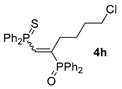	54 [91/9]
9 ^d^	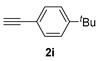	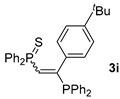	62 ^e^	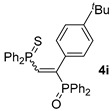	65 [99/1]
10 ^d^	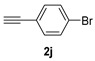	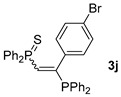	67 [90/10]	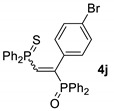	64 [100/0]

^a^ Yields were determined by ^31^P NMR spectroscopy; ^b^ isolated yields; ^c^ reaction time: 9 h; ^d^ reaction time: 2 h. ^e^ Peaks of stereoisomers in ^31^P NMR overlapped.

**Table 4:** Optimization of the reaction conditions for the base-catalyzed double-bond isomerization of **5a**.

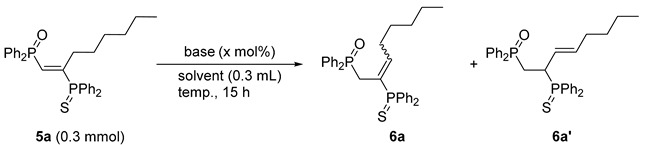					
**Entry**	**Base (mol%)**	**Solvent**	**Temp. (°C)**	**Yield (%) ^a^**	
				**6a [*E*/*Z*]**	**6a’**
1	*^n^*Oct–NH_2_ (100)	CH_3_CN	80	90 [87/13]	6
2	*^n^*Oct–NH_2_ (20)	Toluene	110	90 [87/13] (42)	6
3 ^b^	*^n^*Oct–NH_2_ (5)	Toluene	110	29 [83/17]	N. D.
4 ^b^	DBU (5)	Toluene	110	59 [88/12]	39
5	DBU (40)	Toluene	110	15 [93/7]	83 (56)
6	-	Toluene	110	N. D.	N. D.

^a^ Yields were determined by ^31^P NMR spectroscopy; ^b^ Reaction conditions: **5a** (1.2 mmol), base (5 mol%), toluene (1.2 mL), 110 °C, 15 h.

**Table 5:** Optimization of the reaction conditions for the base-catalyzed double-bond isomerization of **5b**.

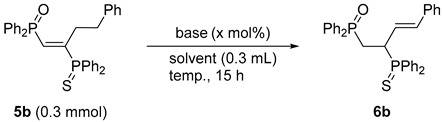				
**Entry**	**Base (mol%)**	**Solvent**	**Temp. (°C)**	**Yield 6b (%) ^a^**
1	*^n^*Oct-NH_2_ (100)	CH_3_CN	80	74
2	*^n^*Bu-NH_2_ (100)	CH_3_CN	80	62
3	*^i^*Pr_2_NH (100)	CH_3_CN	80	4
4	*^i^*Pr_2_NEt (100)	CH_3_CN	80	trace
5	Et_3_N (100)	CH_3_CN	80	2
6	DBU (100)	CH_3_CN	80	94
7	DMAP (100)	CH_3_CN	80	23
8	Cs_2_CO_3_ (100)	CH_3_CN	80	trace
9	*^n^*Oct-NH_2_ (20)	Toluene	110	94
10	DBU (20)	Toluene	110	98
11 ^b^	DBU (5)	Toluene	110	99 (95)

^a^ Yields were determined by ^31^P NMR spectroscopy (isolated yield); ^b^ reaction conditions: **5b** (1.2 mmol), DBU (5 mol%), toluene (1.2 mL), 110 °C, 15 h.

## Data Availability

The data presented in this study are available in the article and in its Supplementary Materials.

## Supplementary Materials

The following are available online. Copies of ^1^H, ^13^C{^1^H}, and ^31^P NMR spectra.

## Sample Availability

Samples of the compounds are not available from the authors.
