# Supplementary material for: Photoinduced Bisphosphination of Alkynes with Phosphorus Interelement Compounds and Its Application to Double-Bond Isomerization
Source: Molecules. 2022 Feb 14;27(4):1284. doi: 10.3390/molecules27041284 (PMC8878596; doi:10.3390/molecules27041284)
Supplement: Supplementary file 1 [file molecules-27-01284-s001.zip › Supplementary Materials_molecules-1576833_.pdf]

molecules

MDPI

Supplementary Materials

Photoinduced Bisphosphination of Alkynes with Phosphorus Interelement Compounds and Its Application to Double-Bond Isomerization

Yuki Yamamoto, Ryo Tanaka, Shintaro Kodama, Akihiro Nomoto and Akiya Ogawa *

Department of Applied Chemistry, Graduate School of Engineering, Osaka Prefecture University, Osaka 599-8531, Japan; syb02137@edu.osakafu-u.ac.jp (Y.Y.); sab02088@edu.osakafu-u.ac.jp (R.T.); skodama@chem.osakafu-u.ac.jp (S.K.); nomoto@chem.osakafu-u.ac.jp (A.N.)

* Correspondence: ogawa@chem.osakafu-u.ac.jp (A.O.)

Contents

Copies of ^{1}H, $^{13}C\{^{1}H\}$, and ^{31}P NMR spectra ···2–25

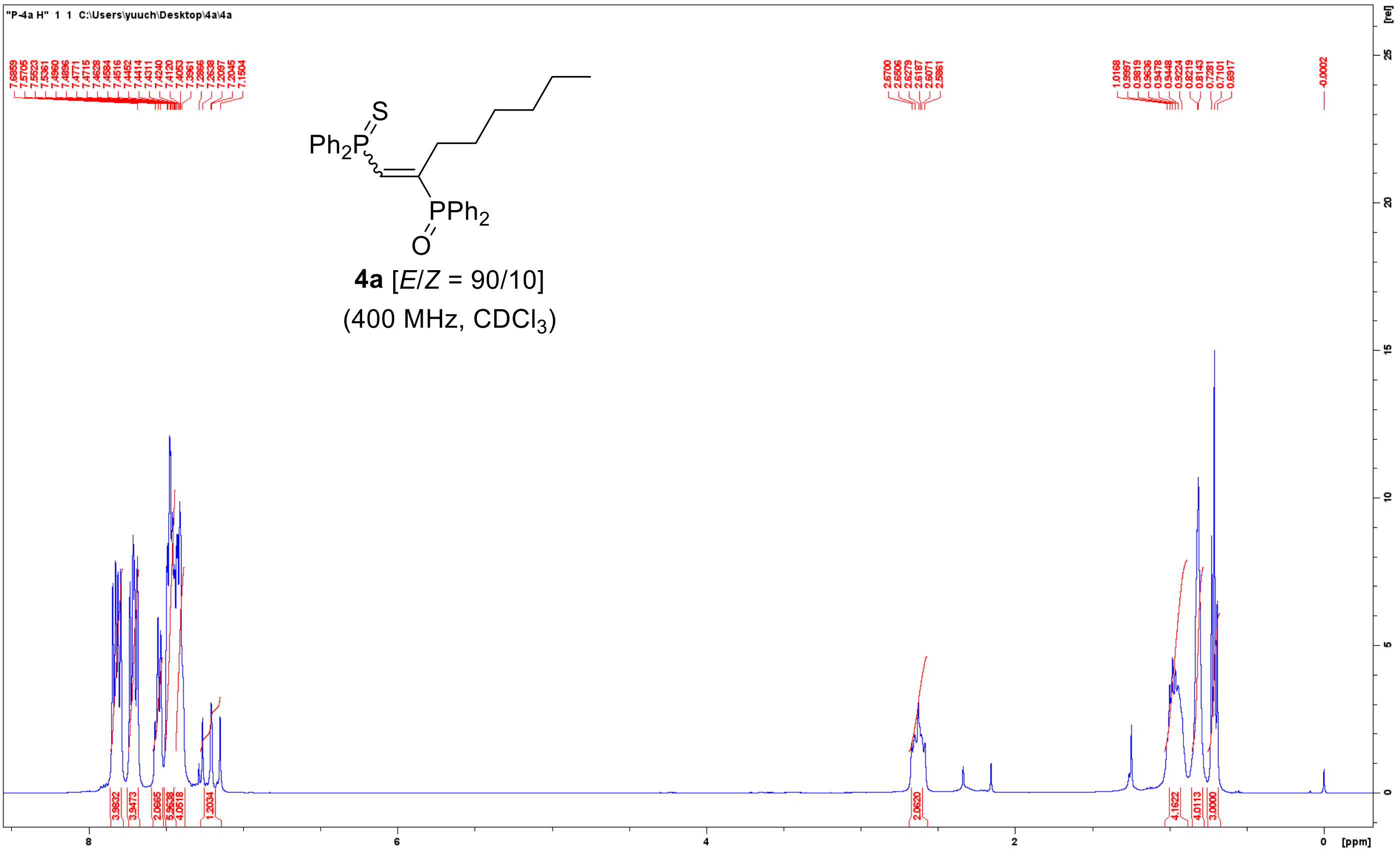

4a [*E*/*Z* = 90/10]
(400 MHz, CDCl3)

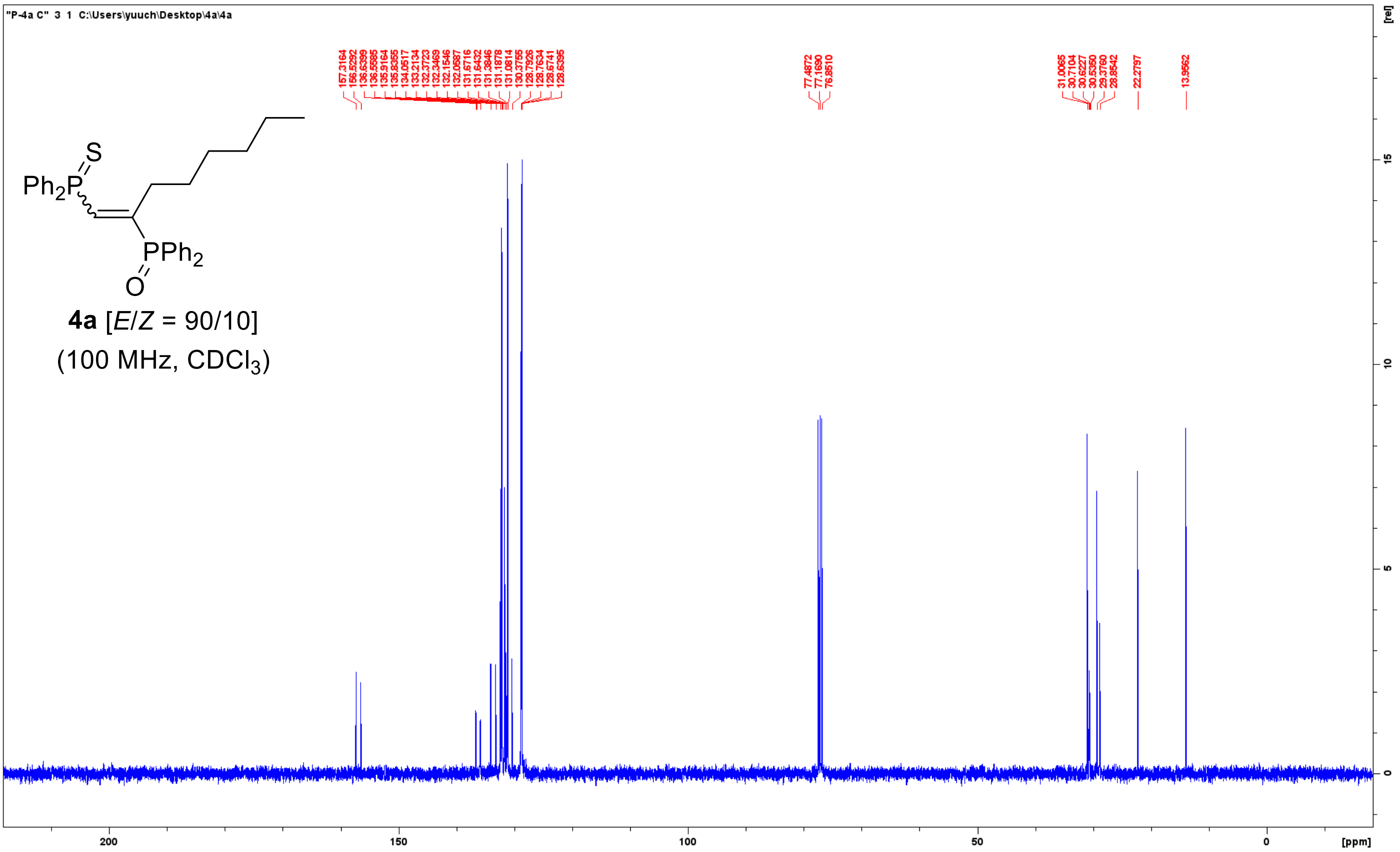

4a [*E*/*Z* = 90/10]
(100 MHz, CDCl3)

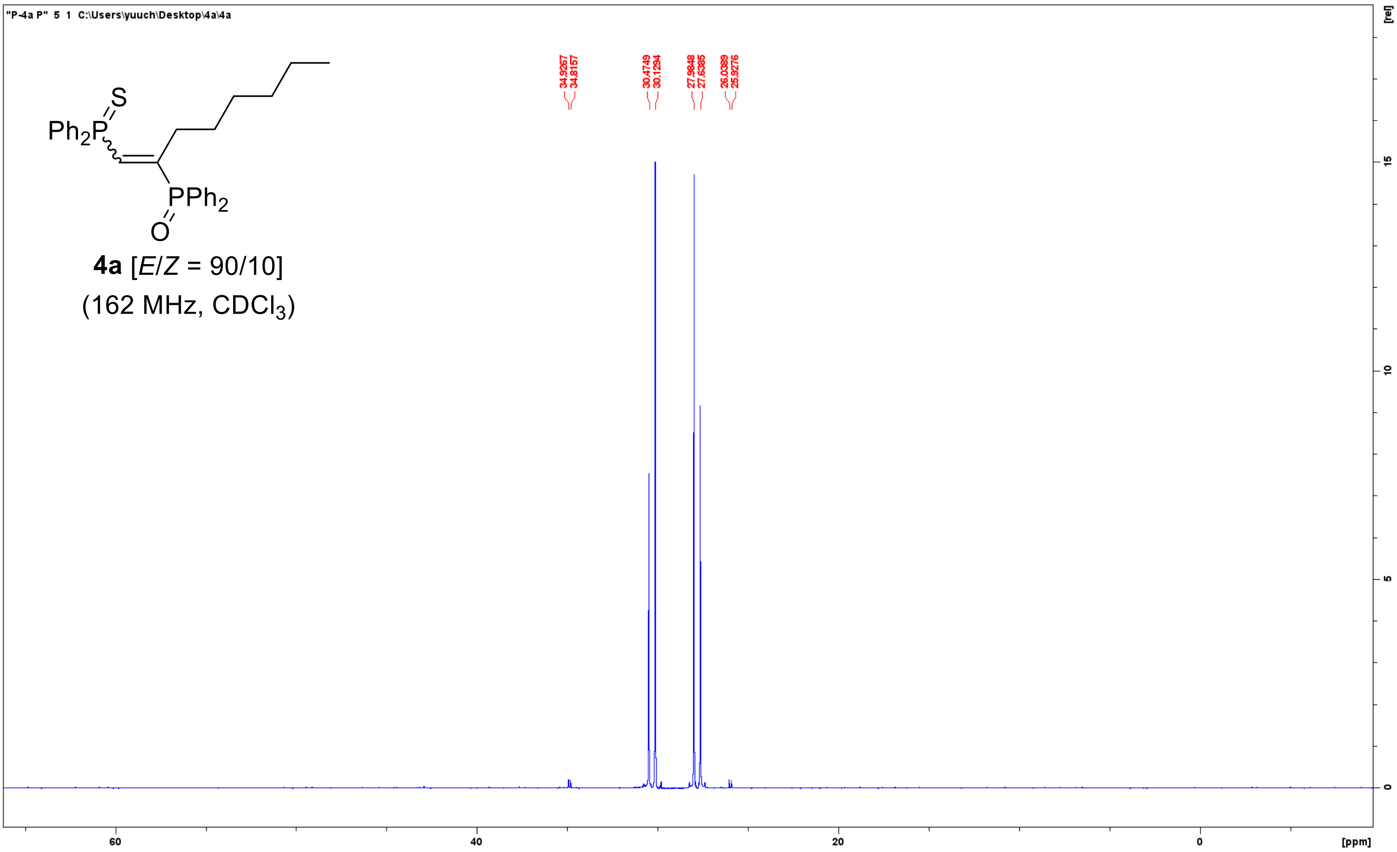
"P-4a P" 5 1 C:\Users\yuuch\Desktop\4a\4a
34.9267
34.8157
30.4749
30.1294
27.9848
27.6385
26.0389
25.9276
S
Ph2P
PPh2
O
4a [E/Z = 90/10]
(162 MHz, CDCl3)
[rel]
15
10
5
0
60
40
20
0
[ppm]

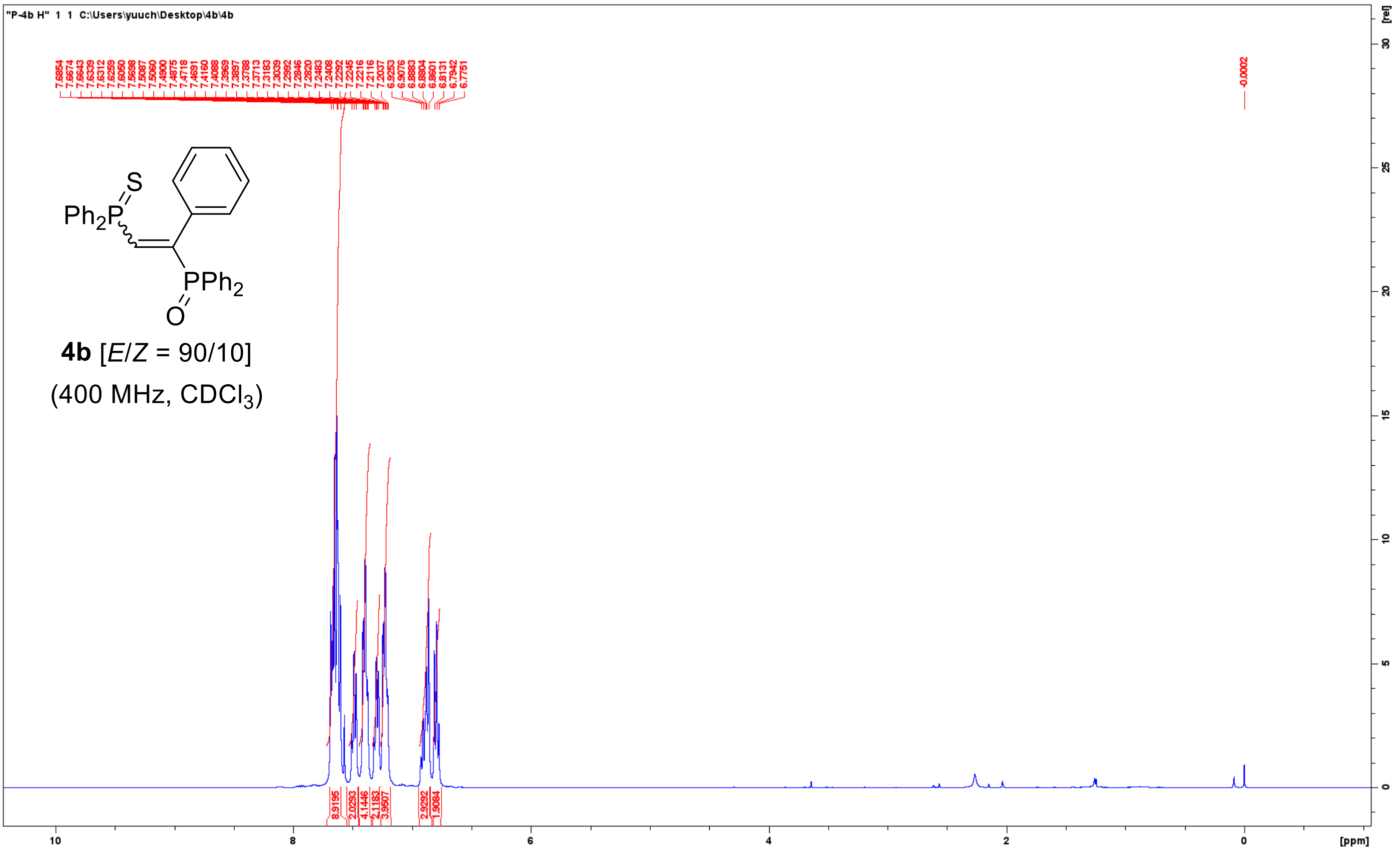
"P-4b H" 1 1 C:\Users\yuuch\Desktop\4b\4b
4b [E/Z = 90/10]
(400 MHz, CDCl3)
[ppm]
[rel]

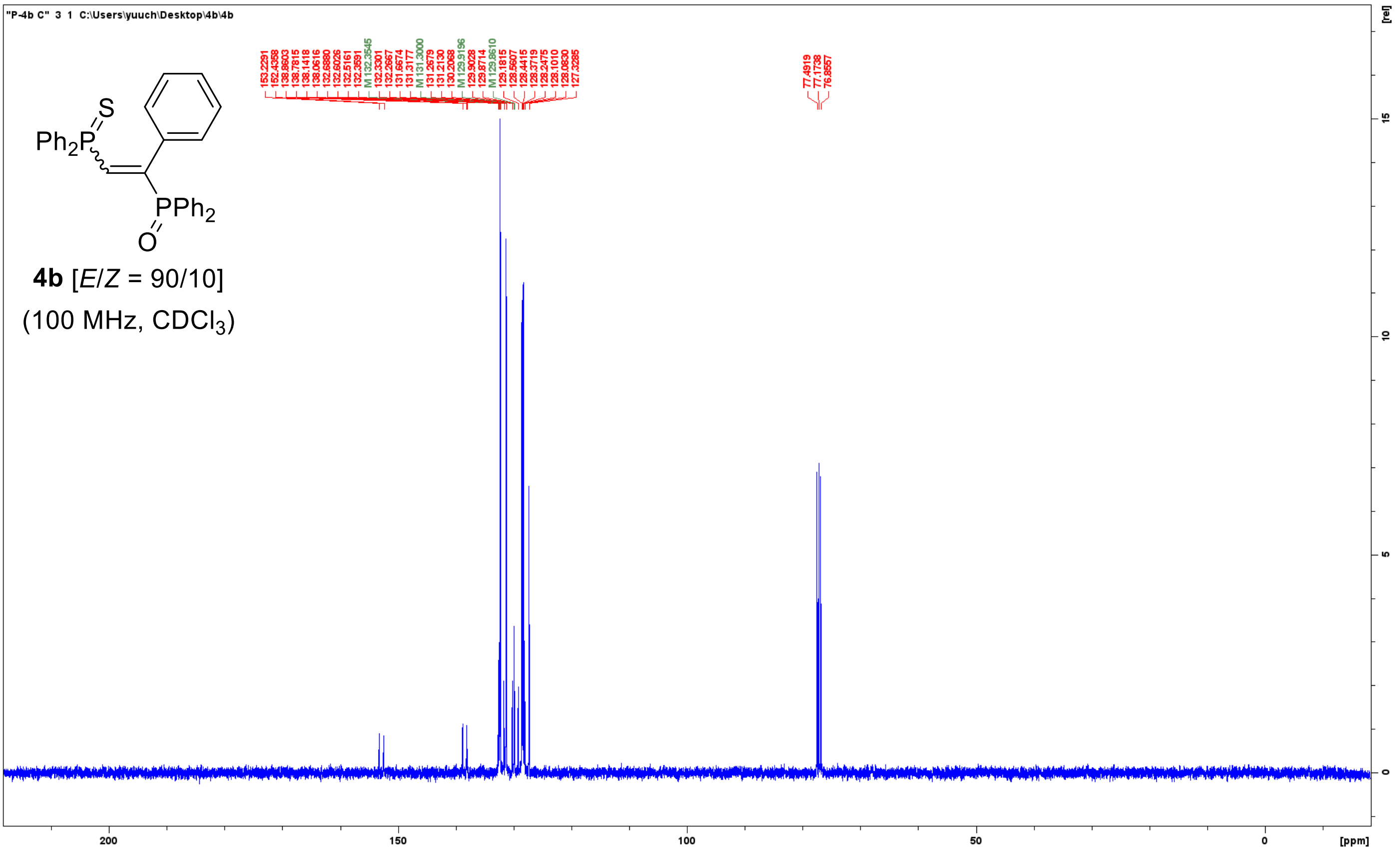
"P-4b C" 3 1 C:\Users\yuuch\Desktop\4b\4b
4b [E/Z = 90/10]
(100 MHz, CDCl3)
[ppm]
[rel]

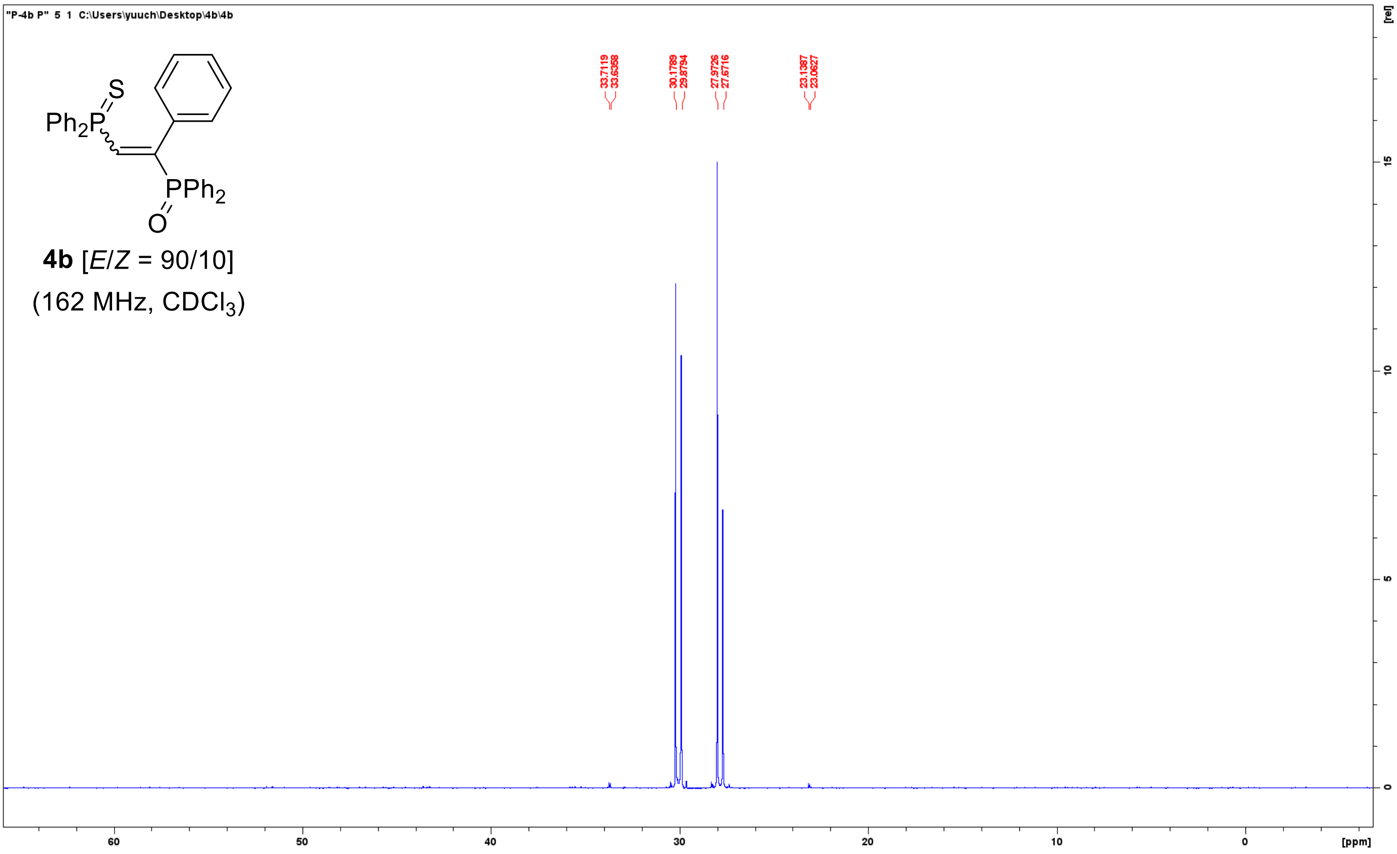
"P-4b P" 5 1 C:\Users\yuuch\Desktop\4b\4b
Ph2P
S
PPh2
O
4b [E/Z = 90/10]
(162 MHz, CDCl3)
33.7119
33.6358
30.1789
29.8794
27.9726
27.6716
23.1387
23.0627
[rel]
15
10
5
0
60
50
40
30
20
10
0
[ppm]

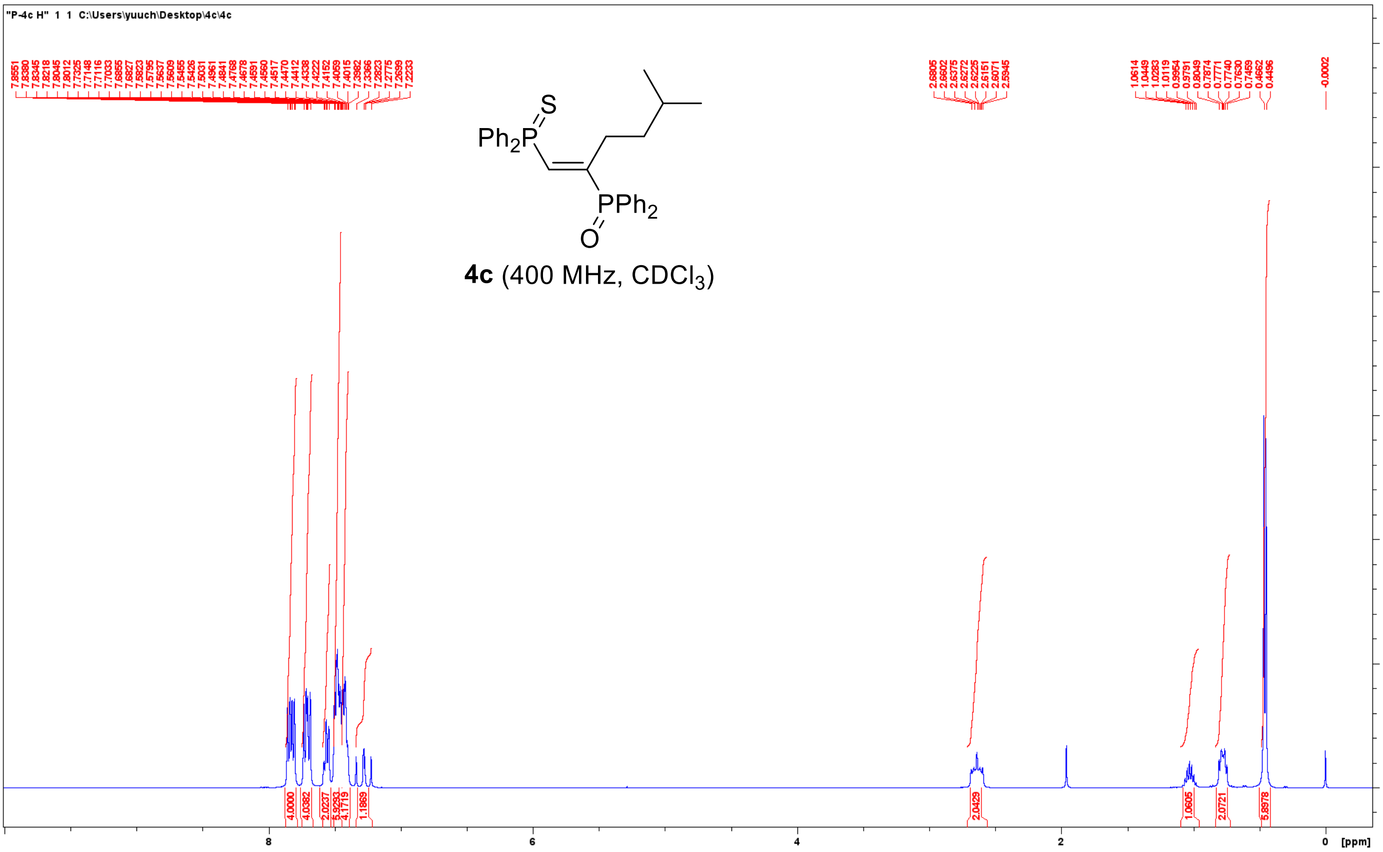
"P-4c H" 1 1 C:\Users\yuuch\Desktop\4c\4c
Ph2P=S
PPh2=O
4c (400 MHz, CDCl3)
[ppm]
[rel]

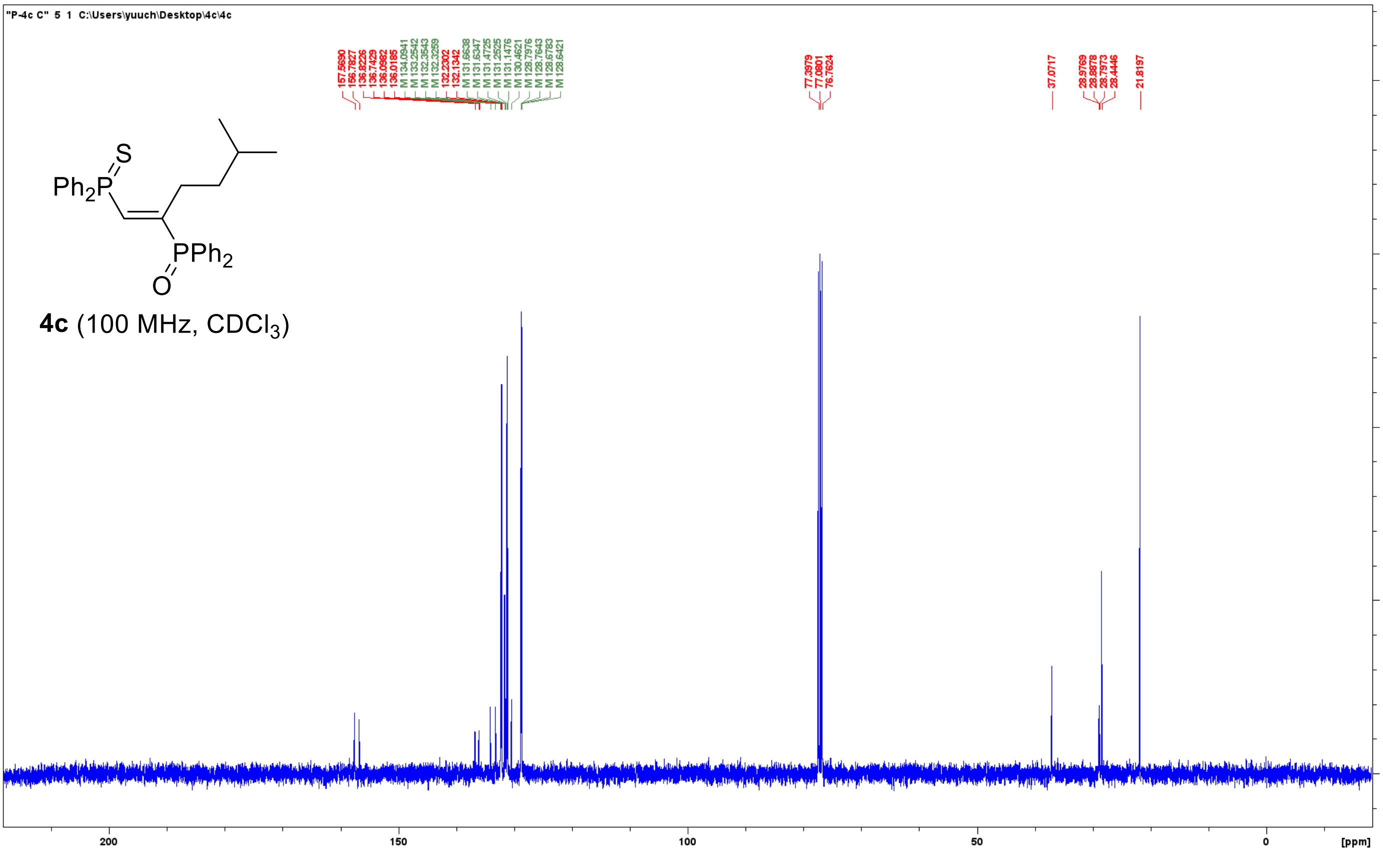
"P-4c C" 5 1 C:\Users\yuuch\Desktop\4c\4c
Ph2P=S
PPh2=O
4c (100 MHz, CDCl3)
[ppm]
[rel]

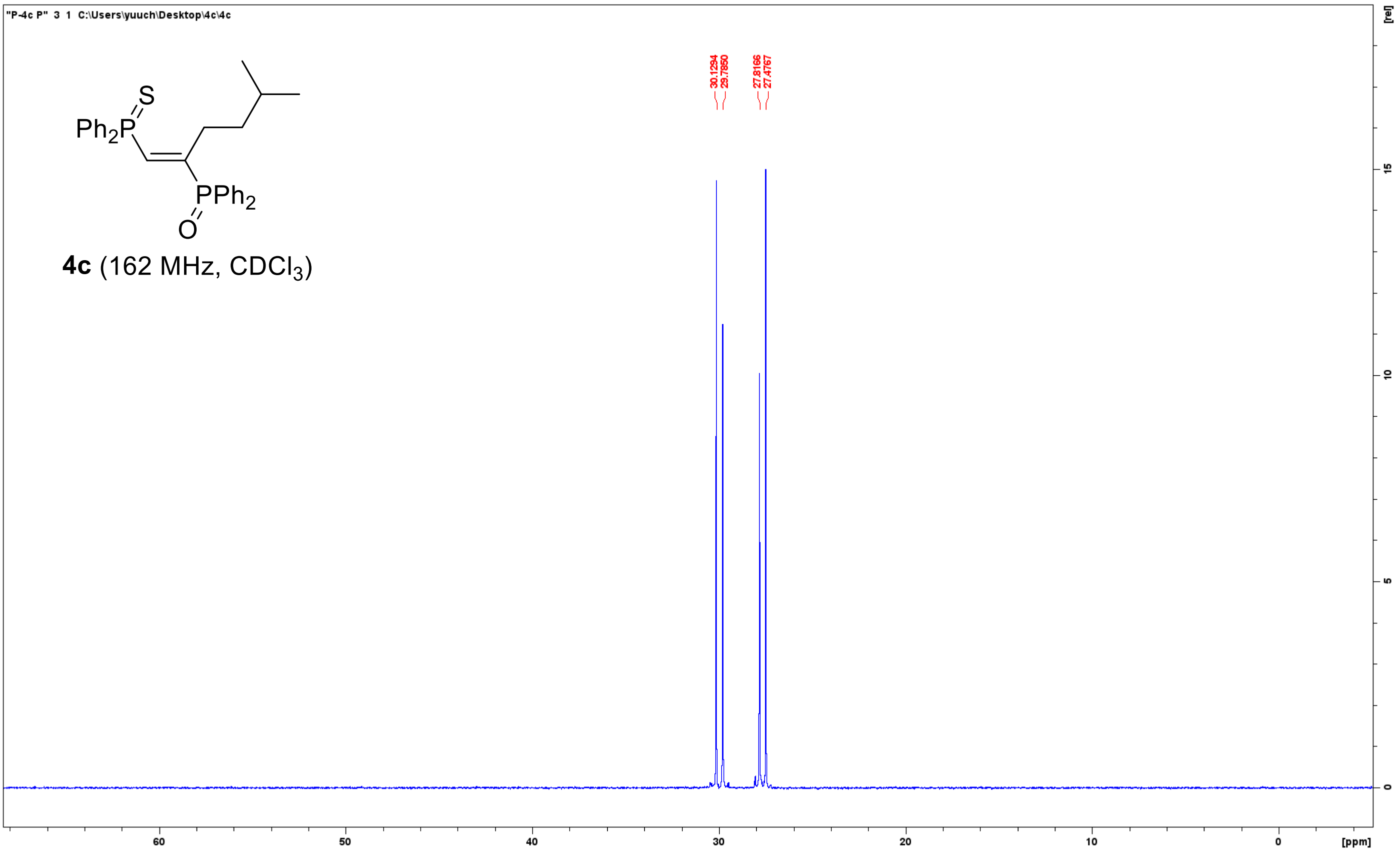
"P-4c P" 3 1 C:\Users\yuuch\Desktop\4c\4c
Ph2P=S
PPh2
O
4c (162 MHz, CDCl3)
30.1294
29.7850
27.8166
27.4767
[rel]
15
10
5
0
60
50
40
30
20
10
0
[ppm]

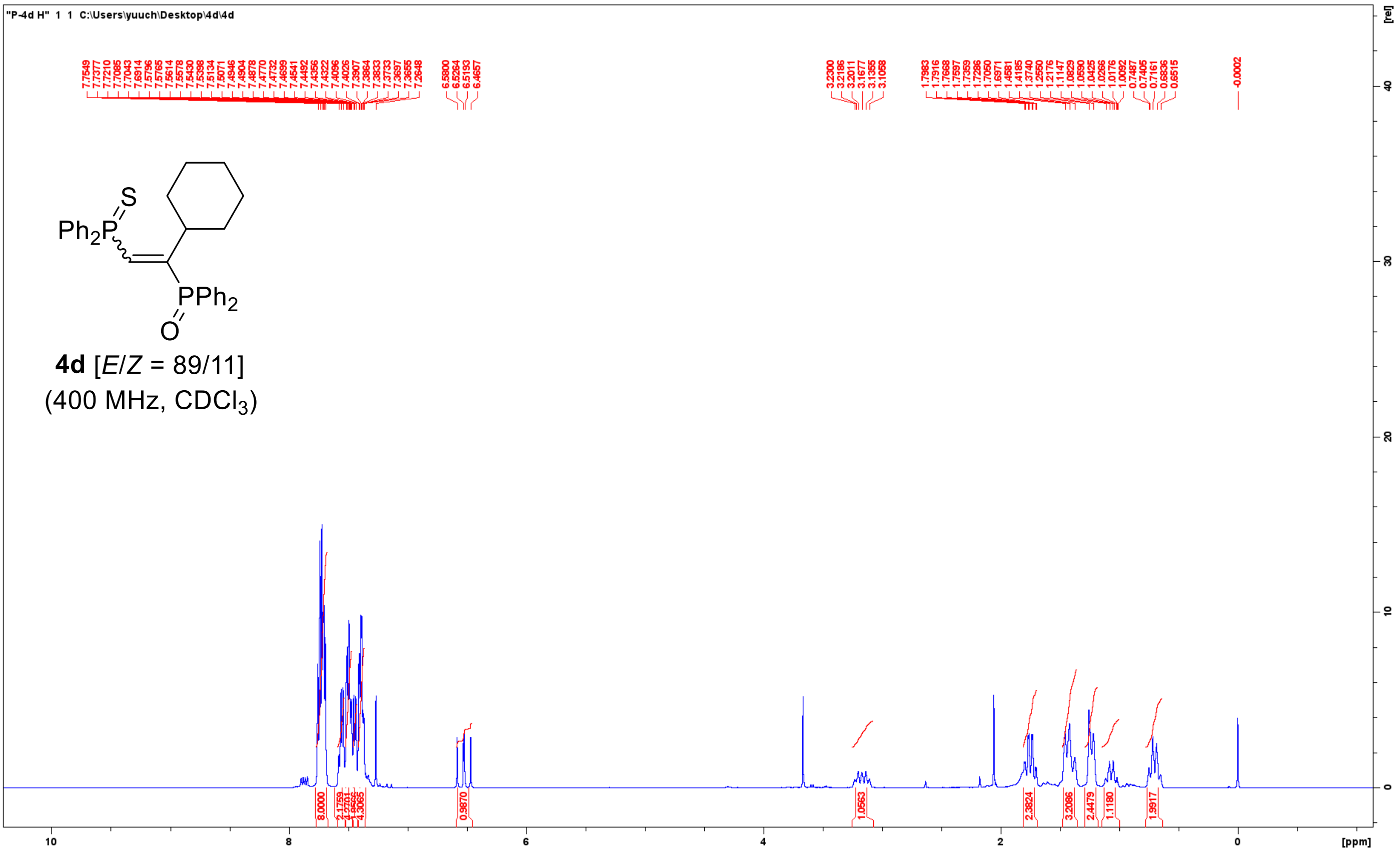
"P-4d H" 1 1 C:\Users\yuuch\Desktop\4d\4d
Ph2P
S
PPh2
O
4d [E/Z = 89/11]
(400 MHz, CDCl3)
[ppm]
[rel]

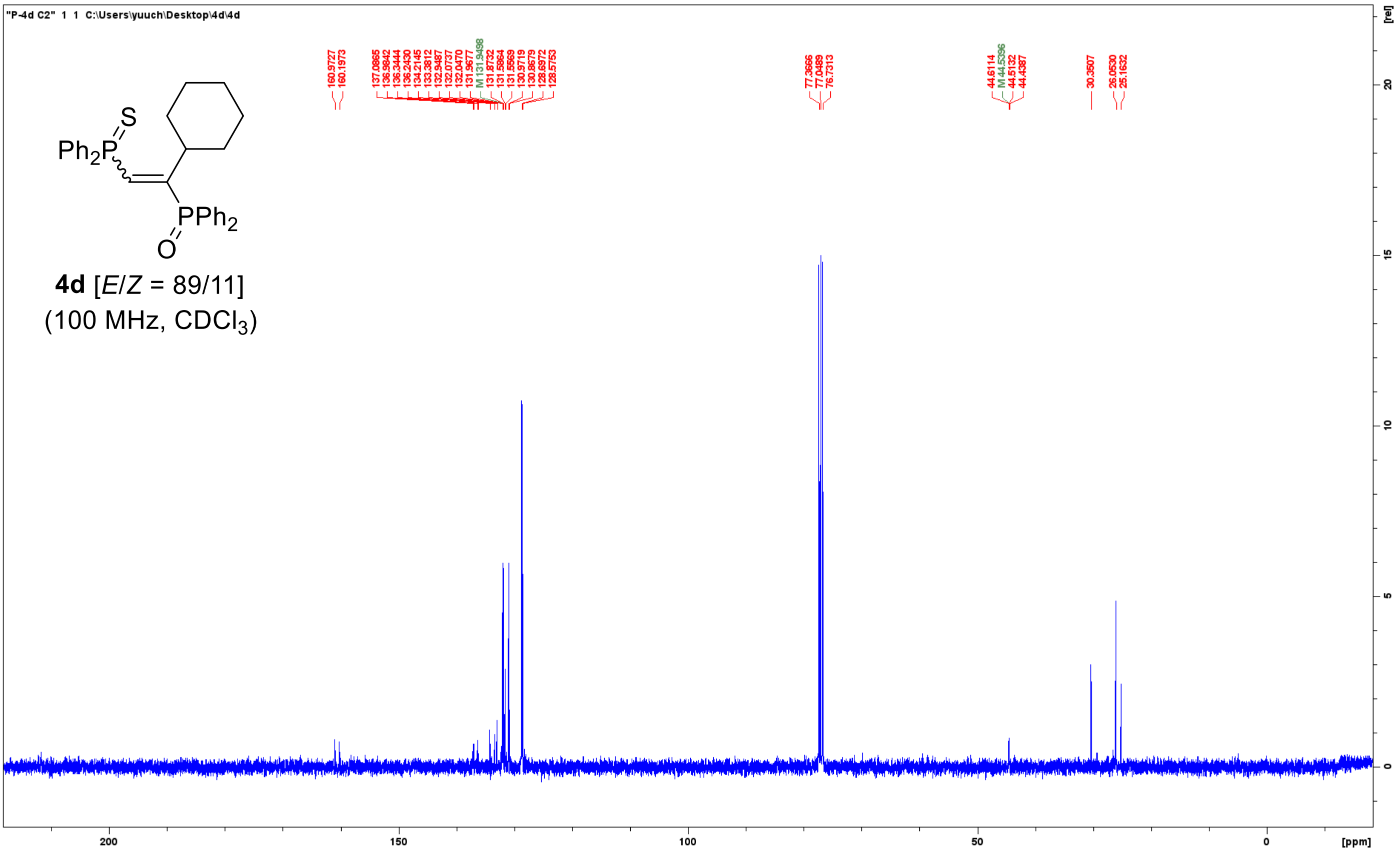
"P-4d C2" 1 1 C:\Users\yuuch\Desktop\4d\4d
Ph2P
S
PPh2
O
4d [E/Z = 89/11]
(100 MHz, CDCl3)
[ppm]
[rel]

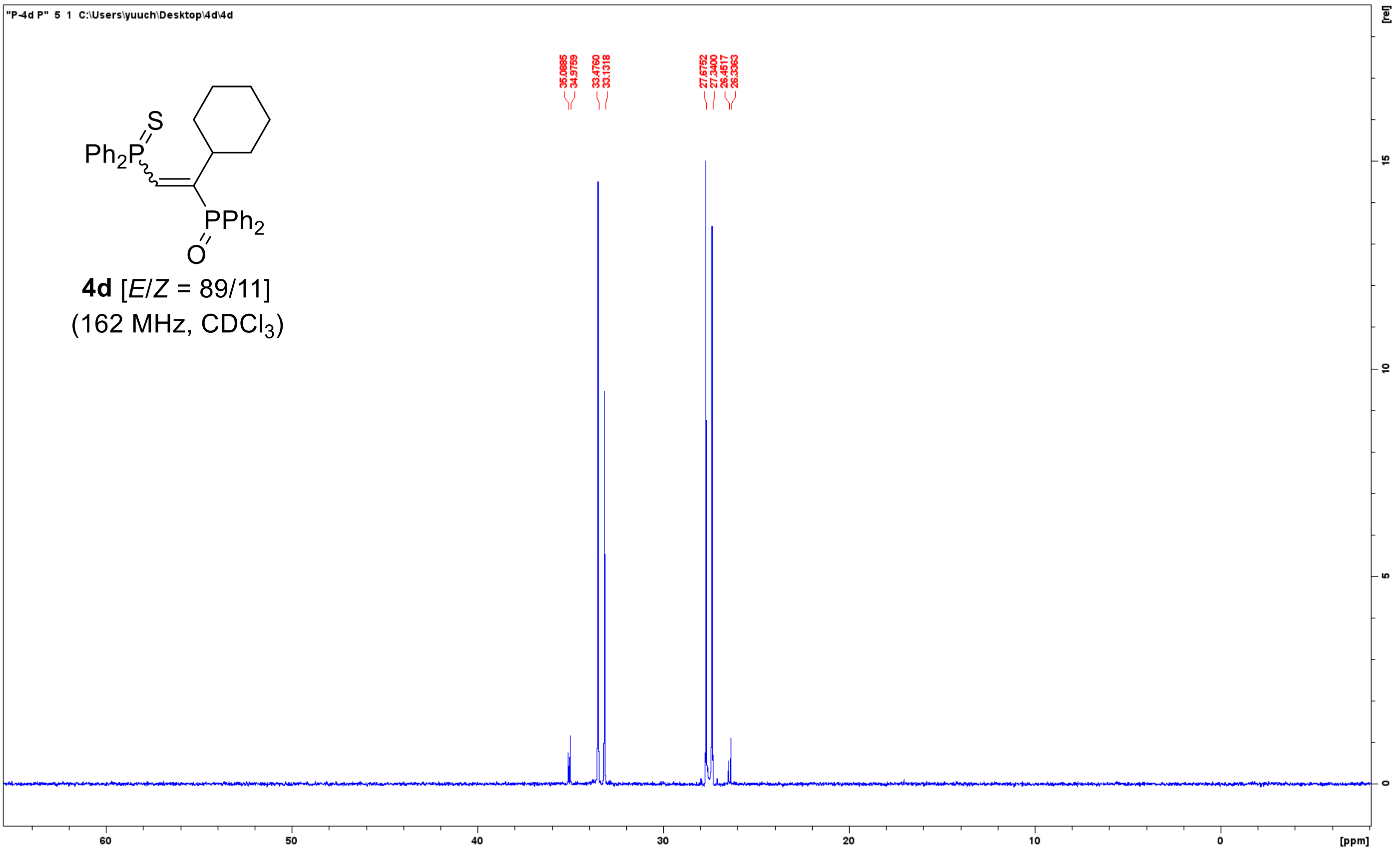
"P-4d P" 5 1 C:\Users\yuuch\Desktop\4d\4d
Ph2P
S
PPh2
O
4d [*E*/*Z* = 89/11]
(162 MHz, CDCl3)
35.0885
34.9759
33.4760
33.1318
27.6752
27.3400
26.4517
26.3363
60
50
40
30
20
10
0
[ppm]
[rel]
15
10
5
0

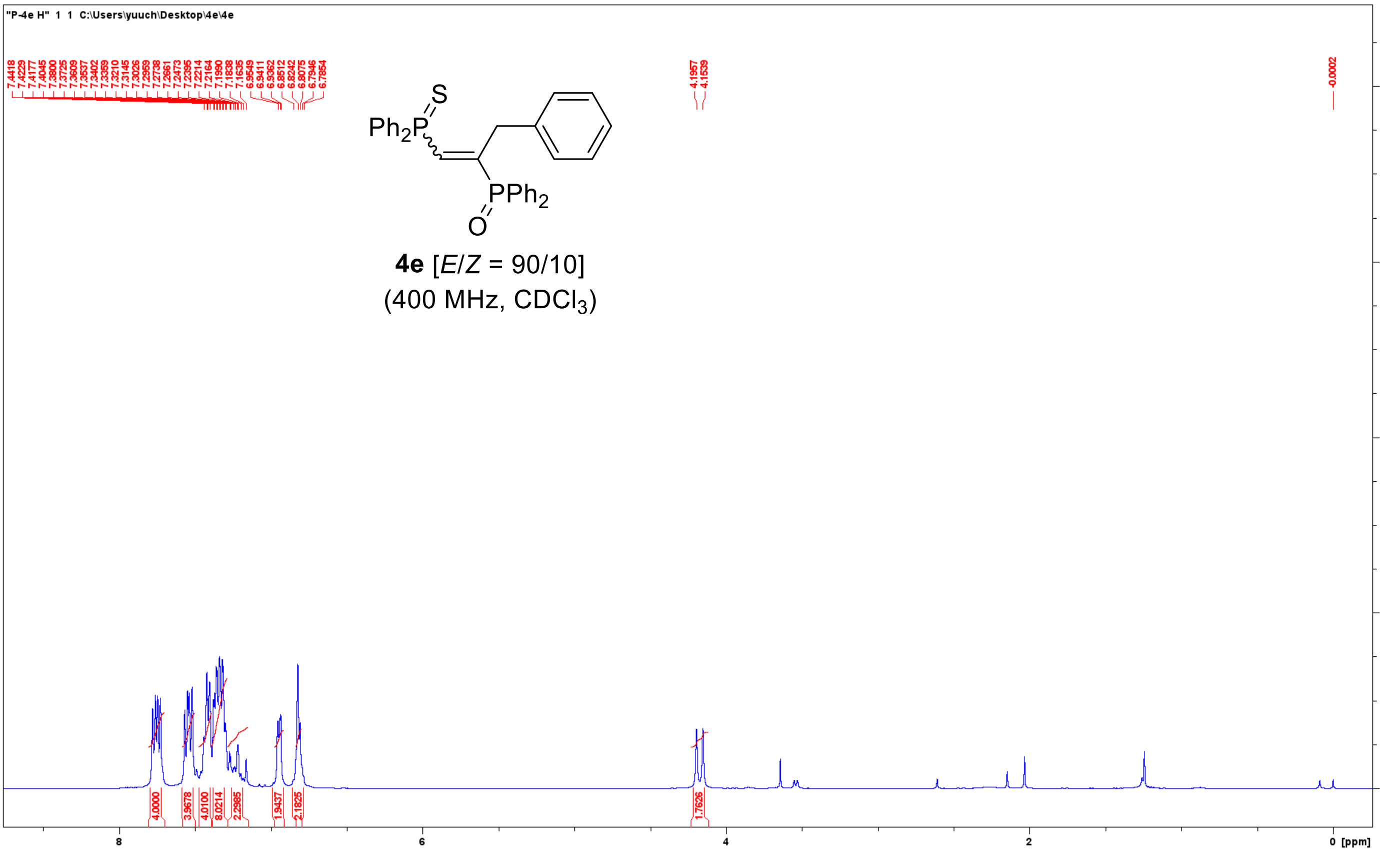
"P-4e H" 1 1 C:\Users\yuuch\Desktop\4e\4e
4e [*E*/*Z* = 90/10]
(400 MHz, $CDCl_3$)
[ppm]
[rel]

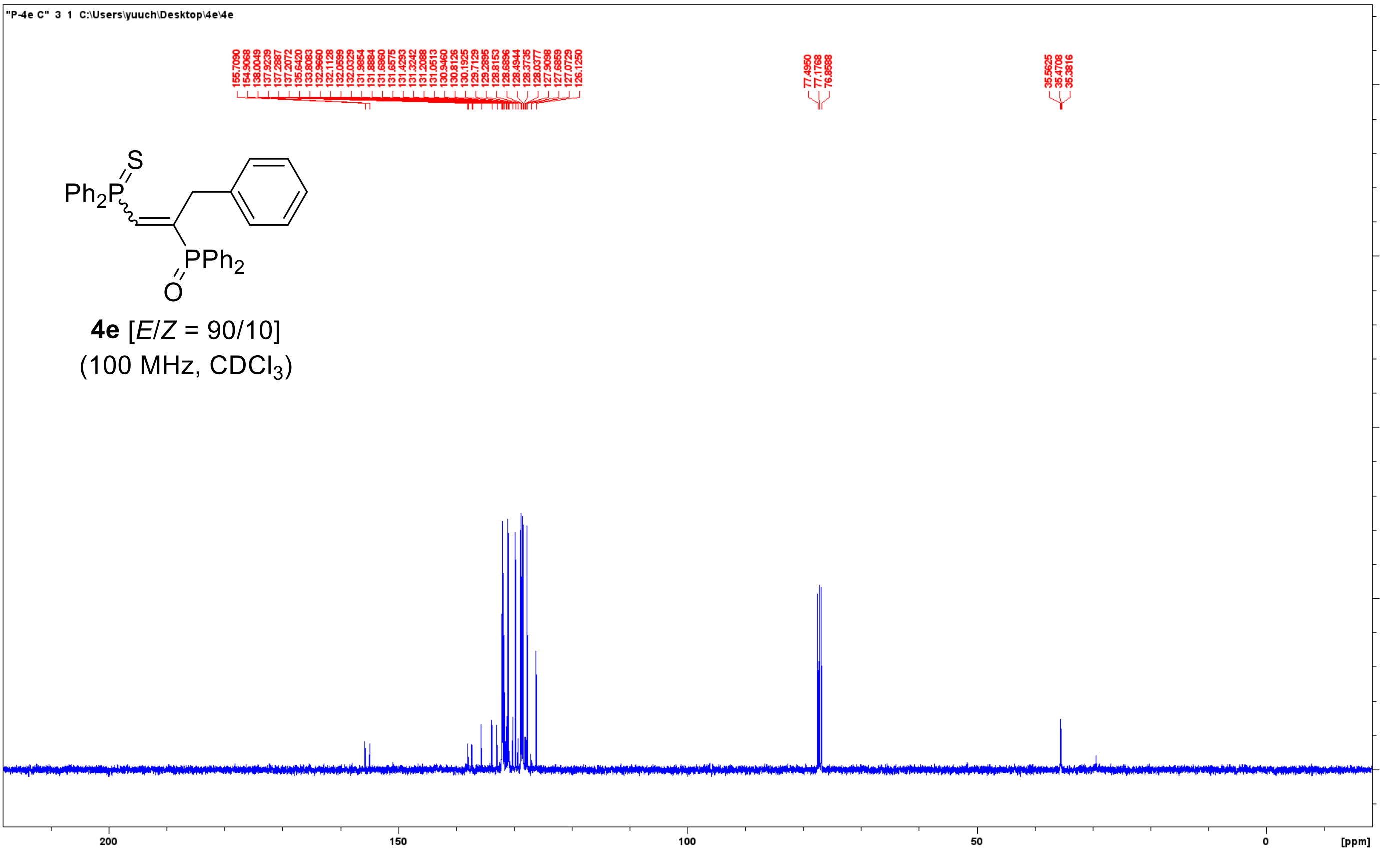
"P-4e C" 3 1 C:\Users\yuuch\Desktop\4e\4e
4e [*E*/*Z* = 90/10]
(100 MHz, $CDCl_3$)
[ppm]
[rel]

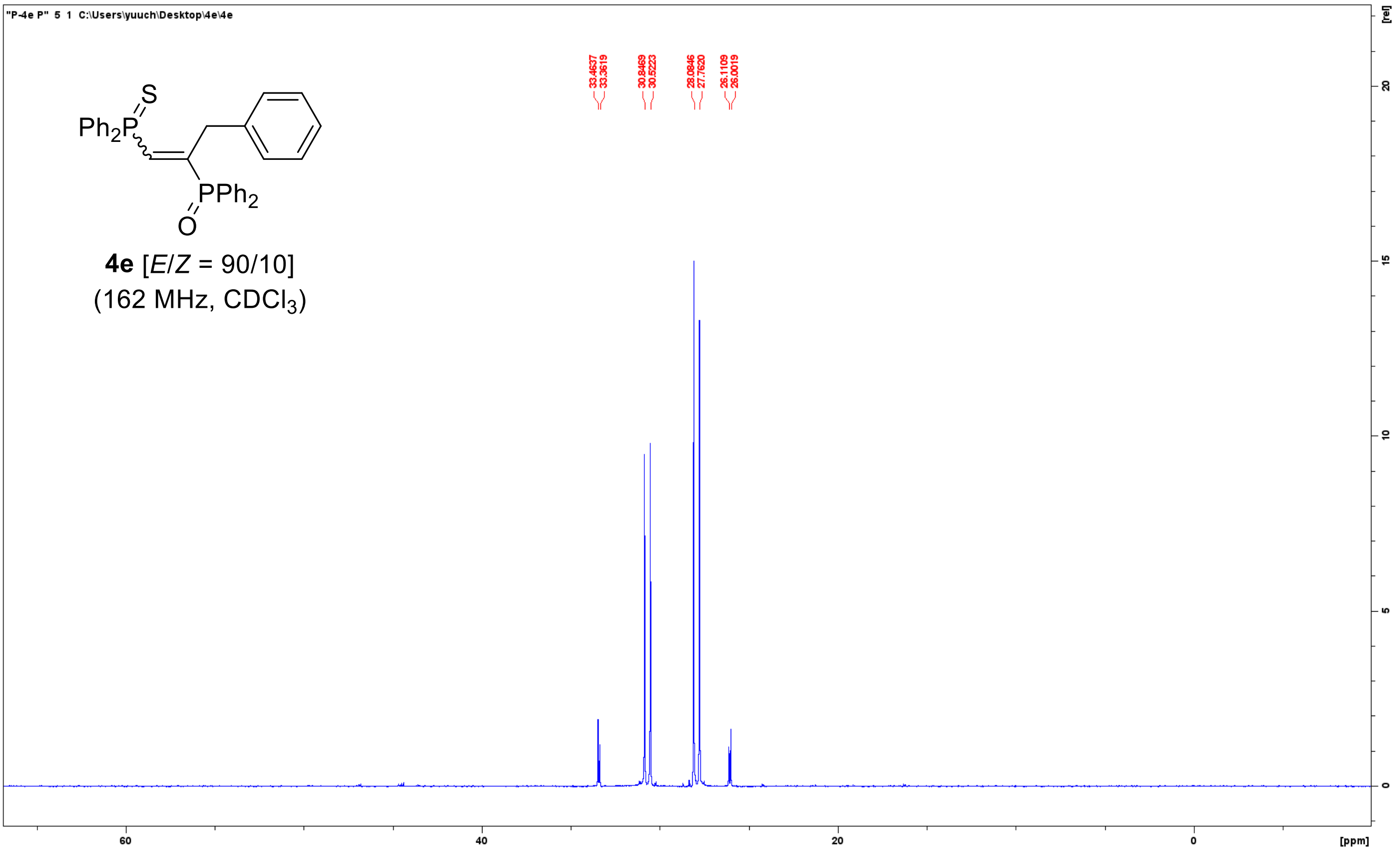
"P-4e P" 5 1 C:\Users\yuuch\Desktop\4e\4e
S
Ph2P
PPh2
O
4e [*E*/*Z* = 90/10]
(162 MHz, CDCl3)
33.4637
33.3619
30.8469
30.5223
28.0846
27.7620
26.1109
26.0019
[rel]
20
15
10
5
0
60
40
20
0
[ppm]

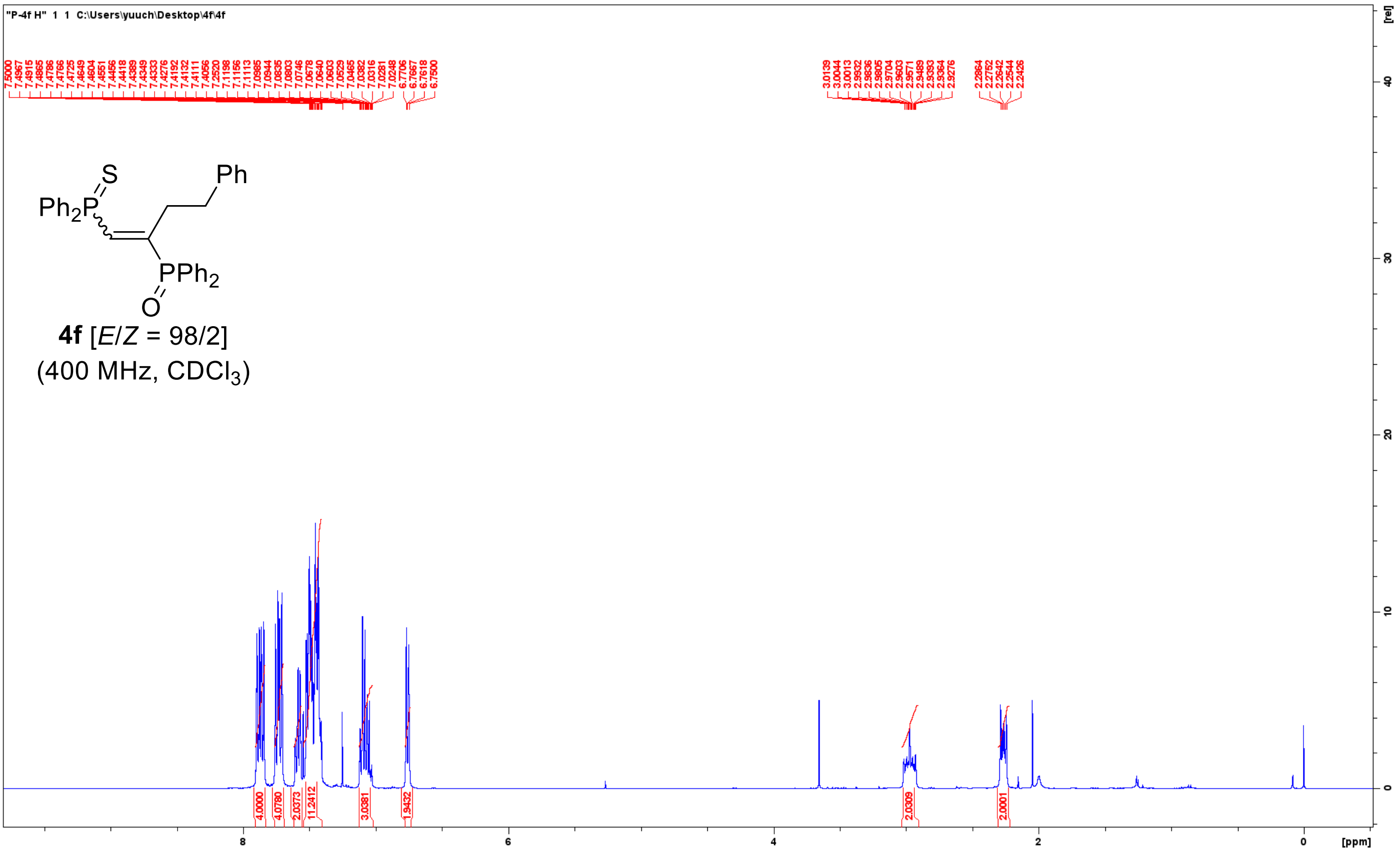
"P-4f H" 1 1 C:\Users\yuuch\Desktop\4f\4f
Ph₂P
S
Ph
PPh₂
O
4f [*E*/*Z* = 98/2]
(400 MHz, CDCl₃)
[ppm]
[rel]

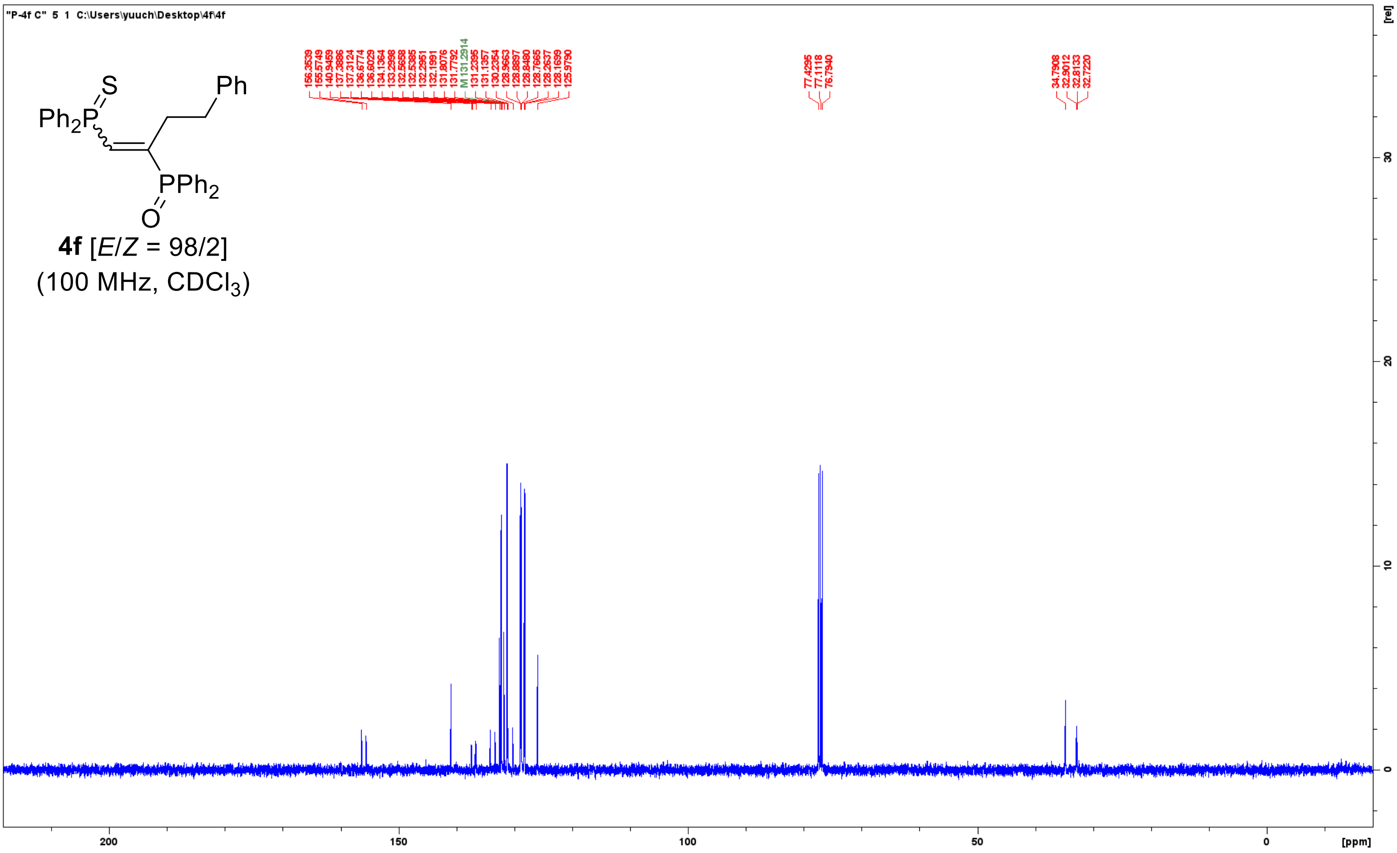
"P-4f C" 5 1 C:\Users\yuuch\Desktop\4f\4f
Ph₂P
S
Ph
PPh₂
O
4f [*E*/*Z* = 98/2]
(100 MHz, CDCl₃)
[ppm]
[rel]

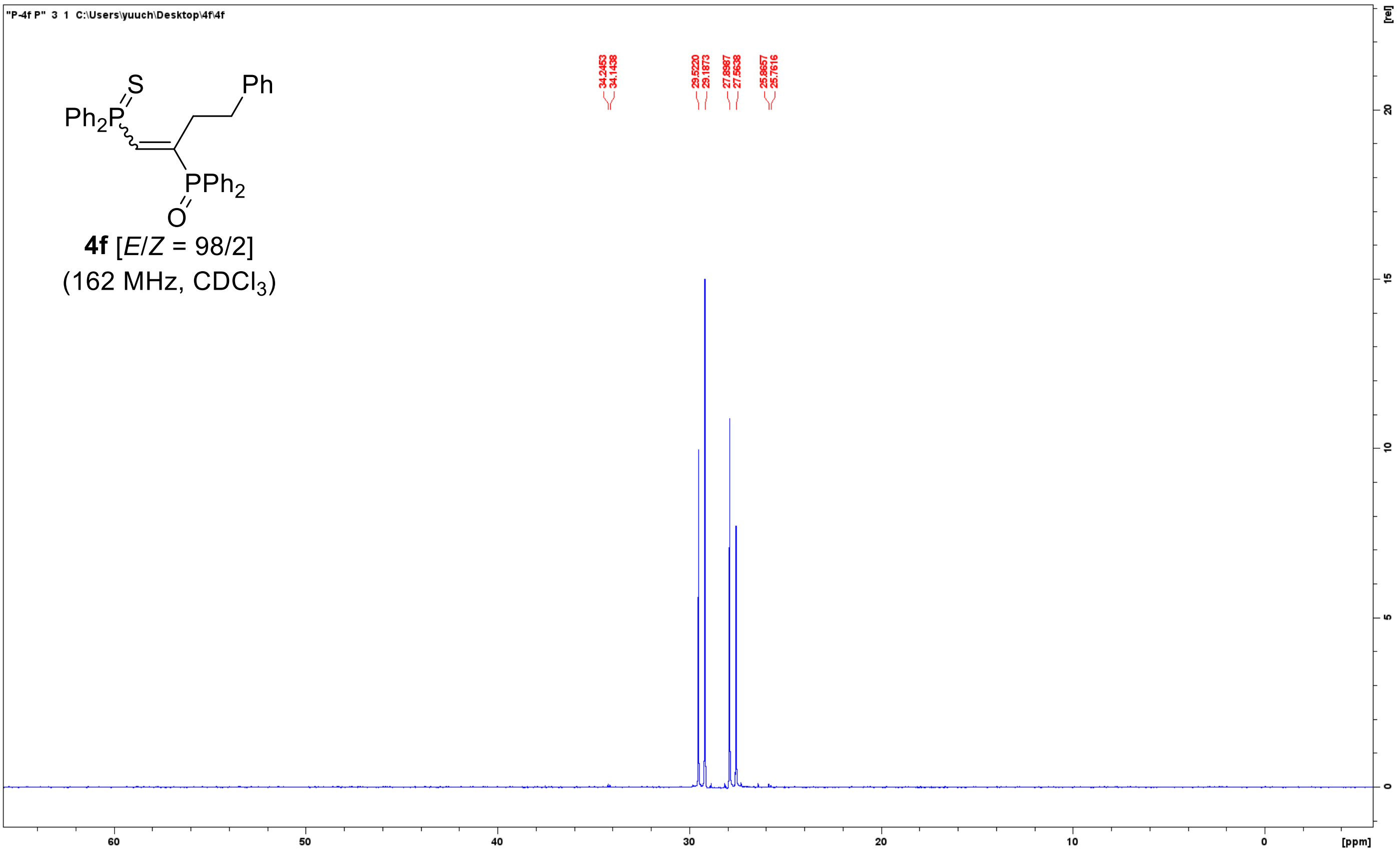
"P-4f P" 3 1 C:\Users\yuuch\Desktop\4f\4f
Ph2P
S
Ph
PPh2
O
4f [E/Z = 98/2]
(162 MHz, CDCl3)
34.2453
34.1438
29.5220
29.1873
27.8987
27.5638
25.8657
25.7616
[ppm]
[rel]

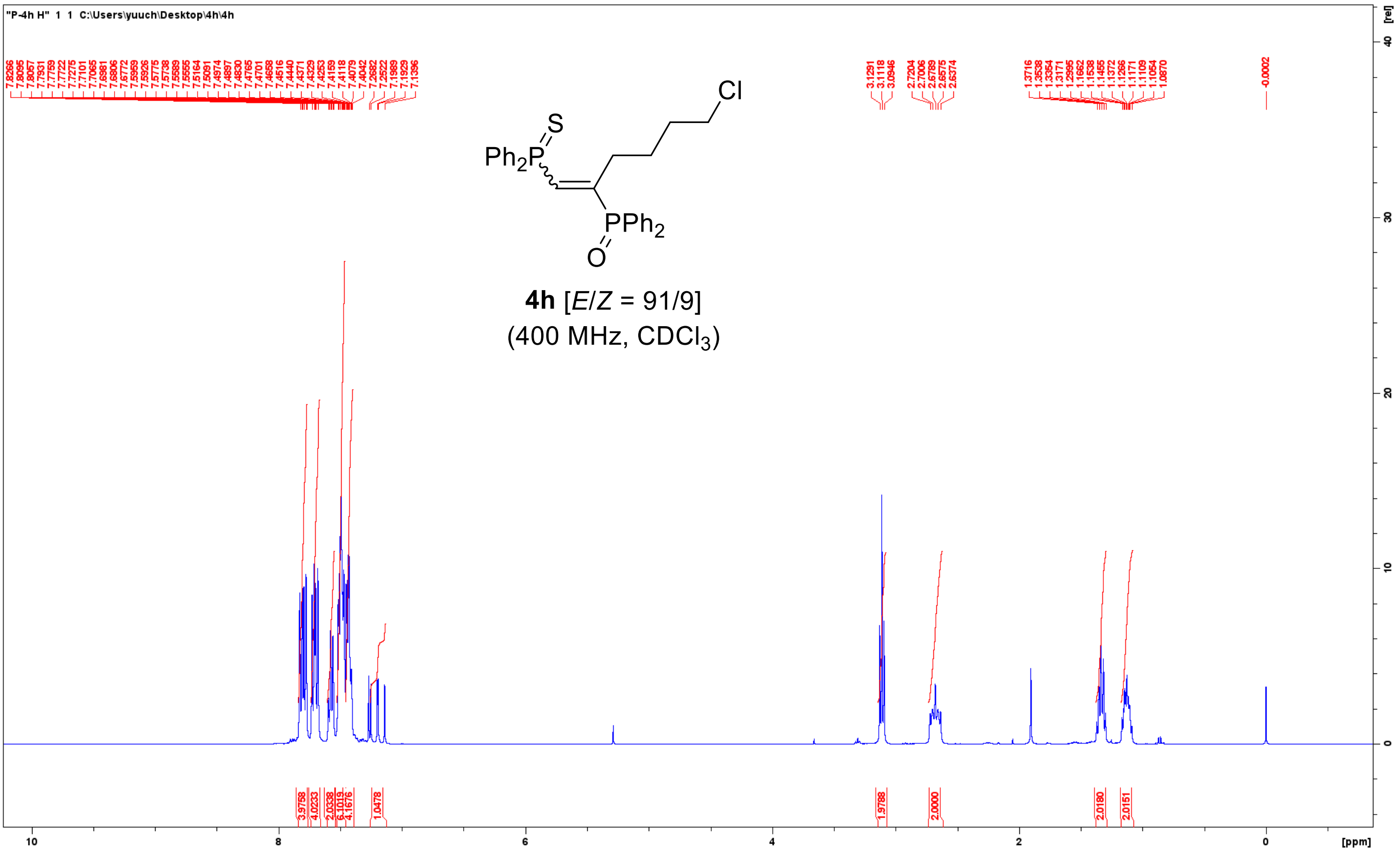
"P-4h H" 1 1 C:\Users\yuuch\Desktop\4h\4h
Cl
S
Ph2P
PPh2
O
4h [E/Z = 91/9]
(400 MHz, CDCl3)
[rel]
[ppm]

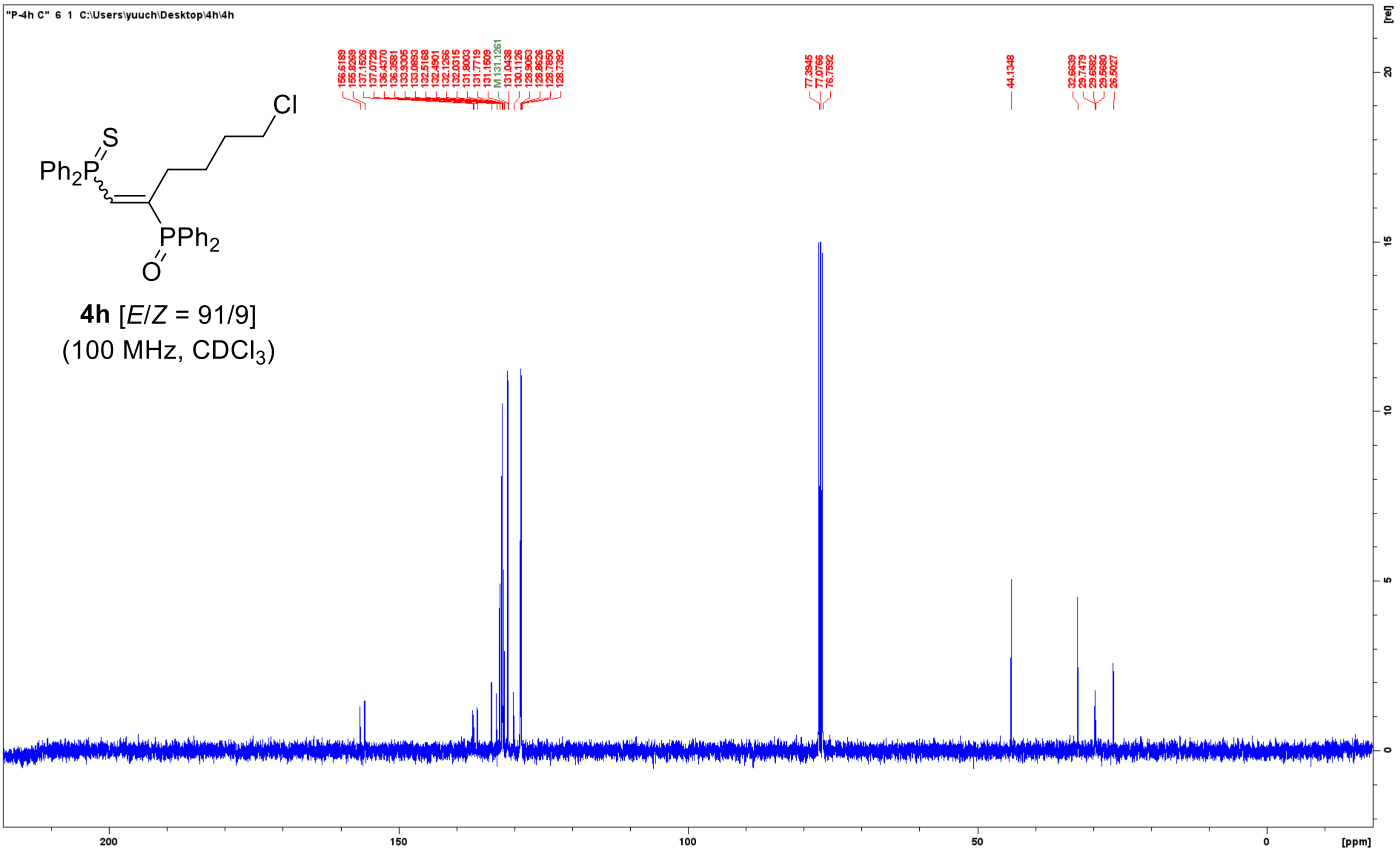
"P-4h C" 6 1 C:\Users\yuuch\Desktop\4h\4h
Cl
S
Ph2P
PPh2
O
4h [E/Z = 91/9]
(100 MHz, CDCl3)
[rel]
[ppm]

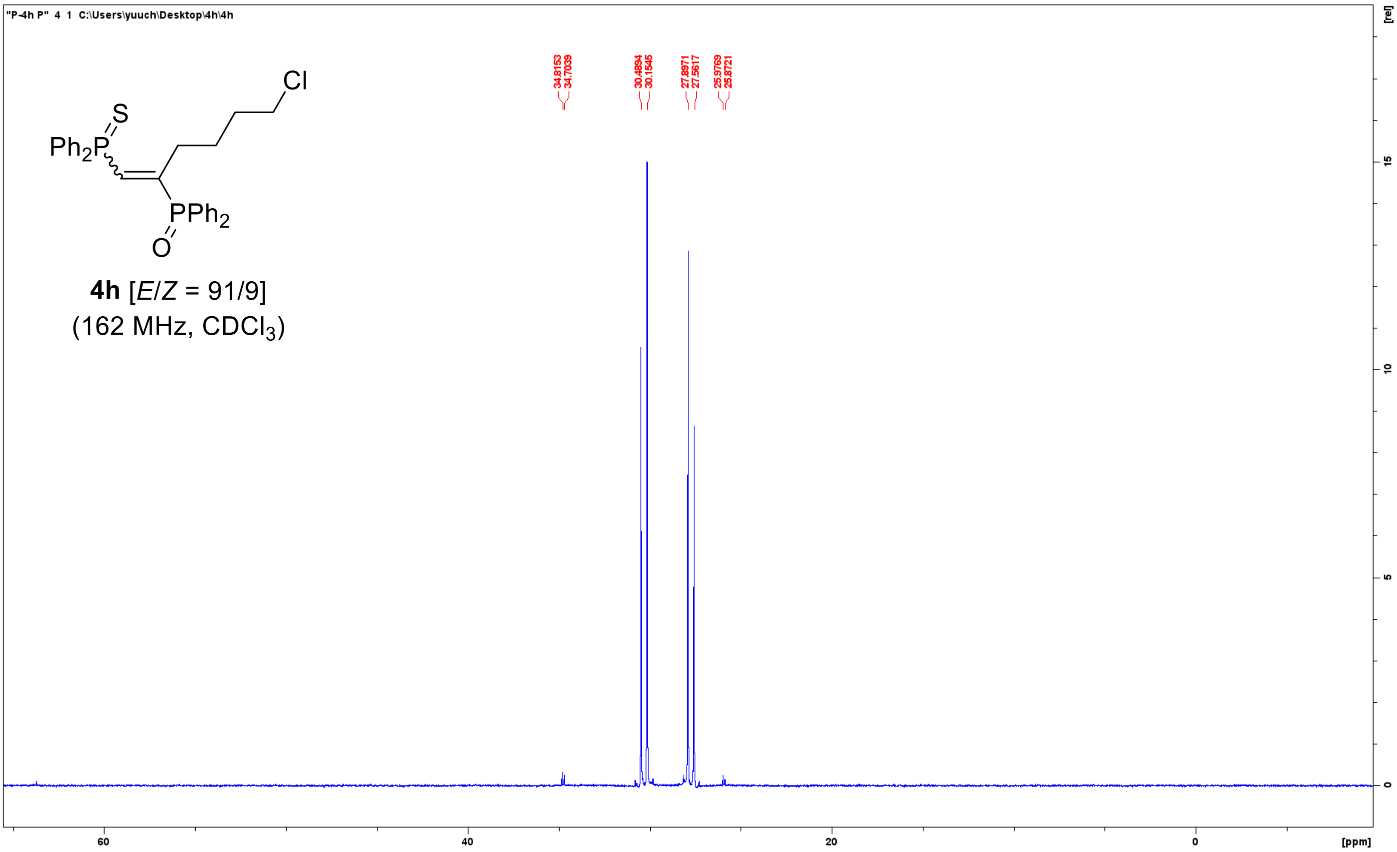
"P-4h P" 4 1 C:\Users\yuuch\Desktop\4h\4h
34.8153
34.7039
30.4894
30.1545
27.8971
27.5617
25.9769
25.8721
Cl
S
Ph2P
PPh2
O
4h [E/Z = 91/9]
(162 MHz, CDCl3)
[rel]
15
10
5
0
60
40
20
0
[ppm]

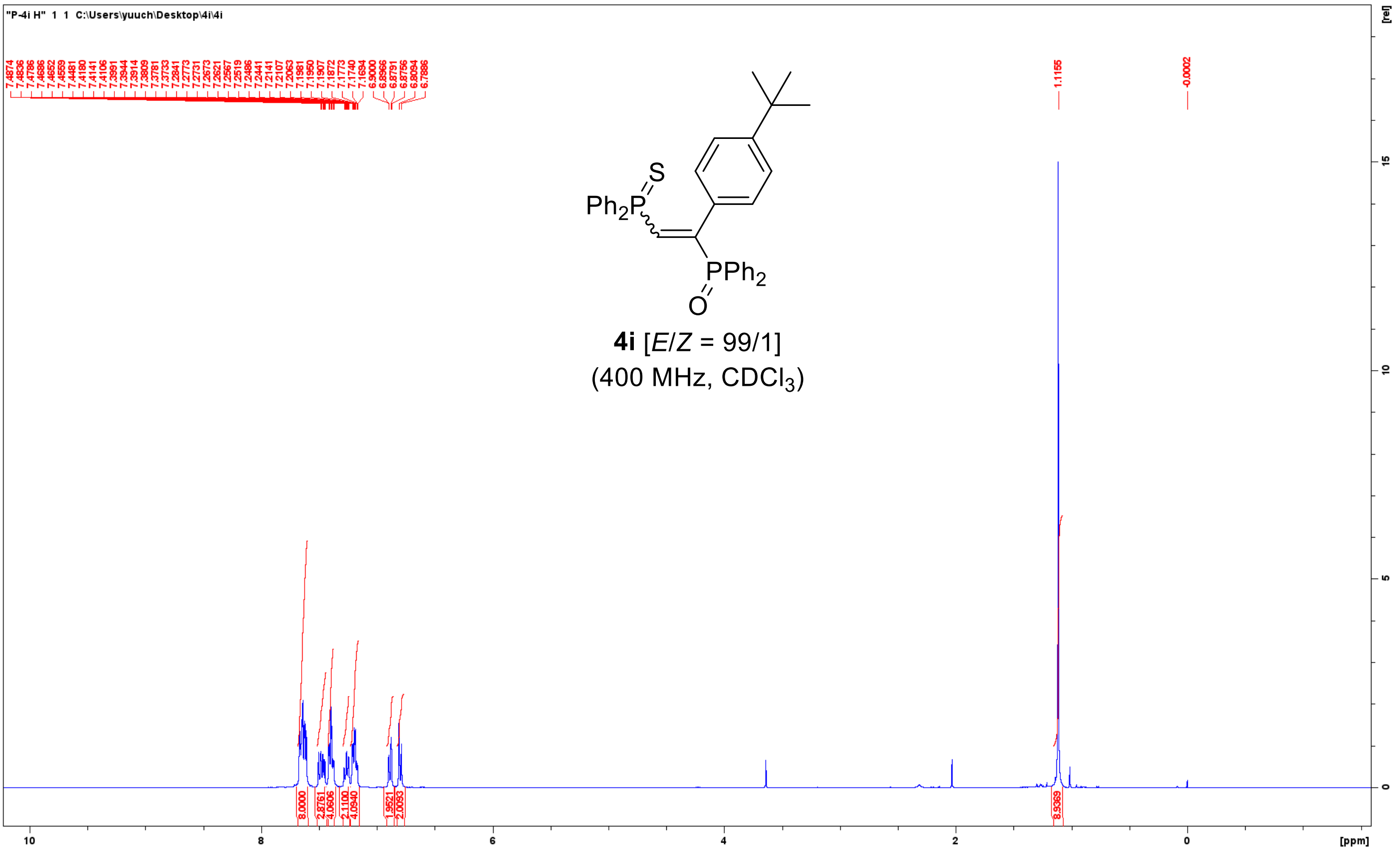
"P-4i H" 1 1 C:\Users\yuuch\Desktop\4i\4i
Ph2P
S
PPh2
O
4i [E/Z = 99/1]
(400 MHz, CDCl3)
[ppm]
[rel]

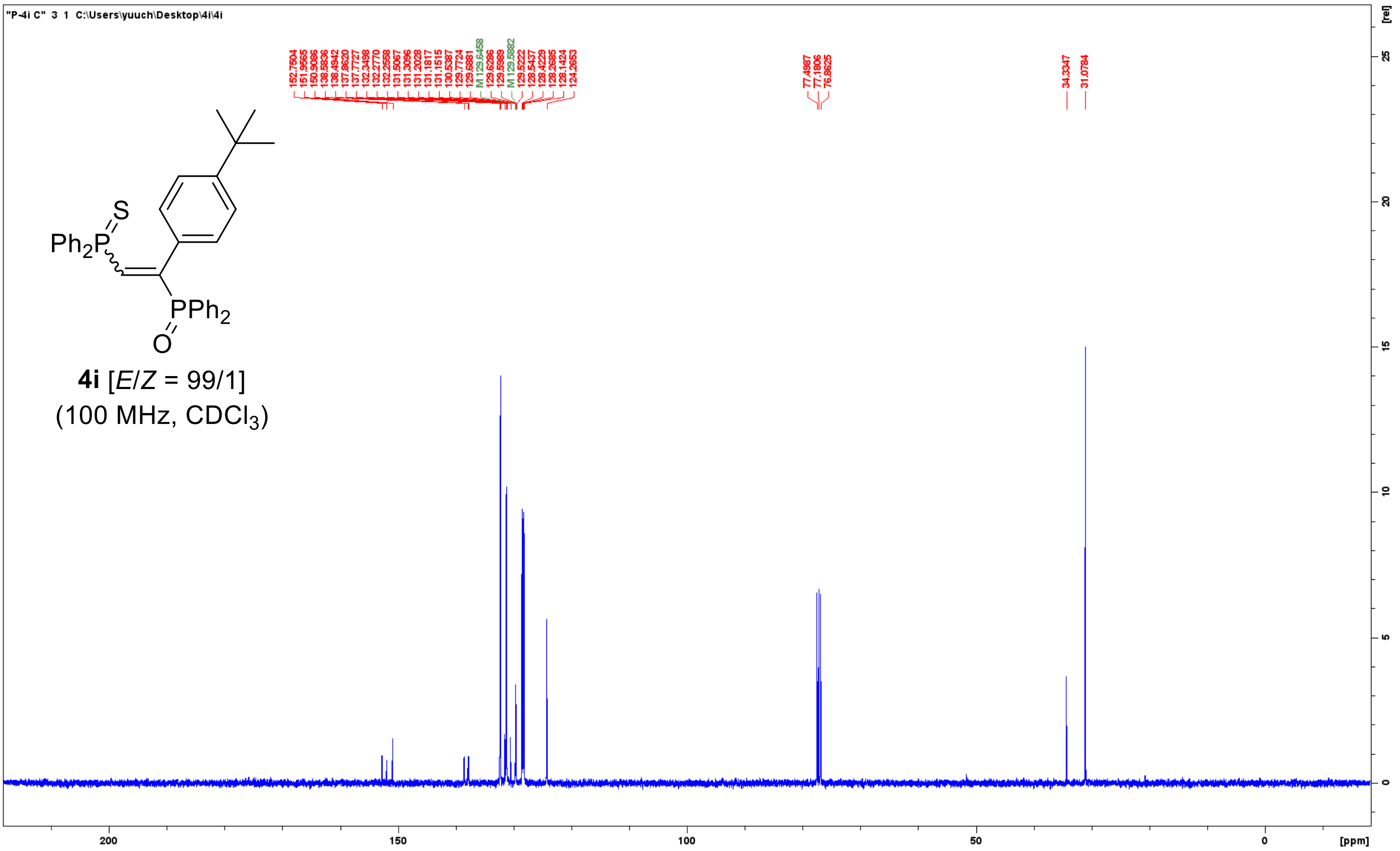
"P-4i C" 3 1 C:\Users\yuuch\Desktop\4i\4i
Ph2P
S
PPh2
O
4i [E/Z = 99/1]
(100 MHz, CDCl3)
[ppm]
[rel]

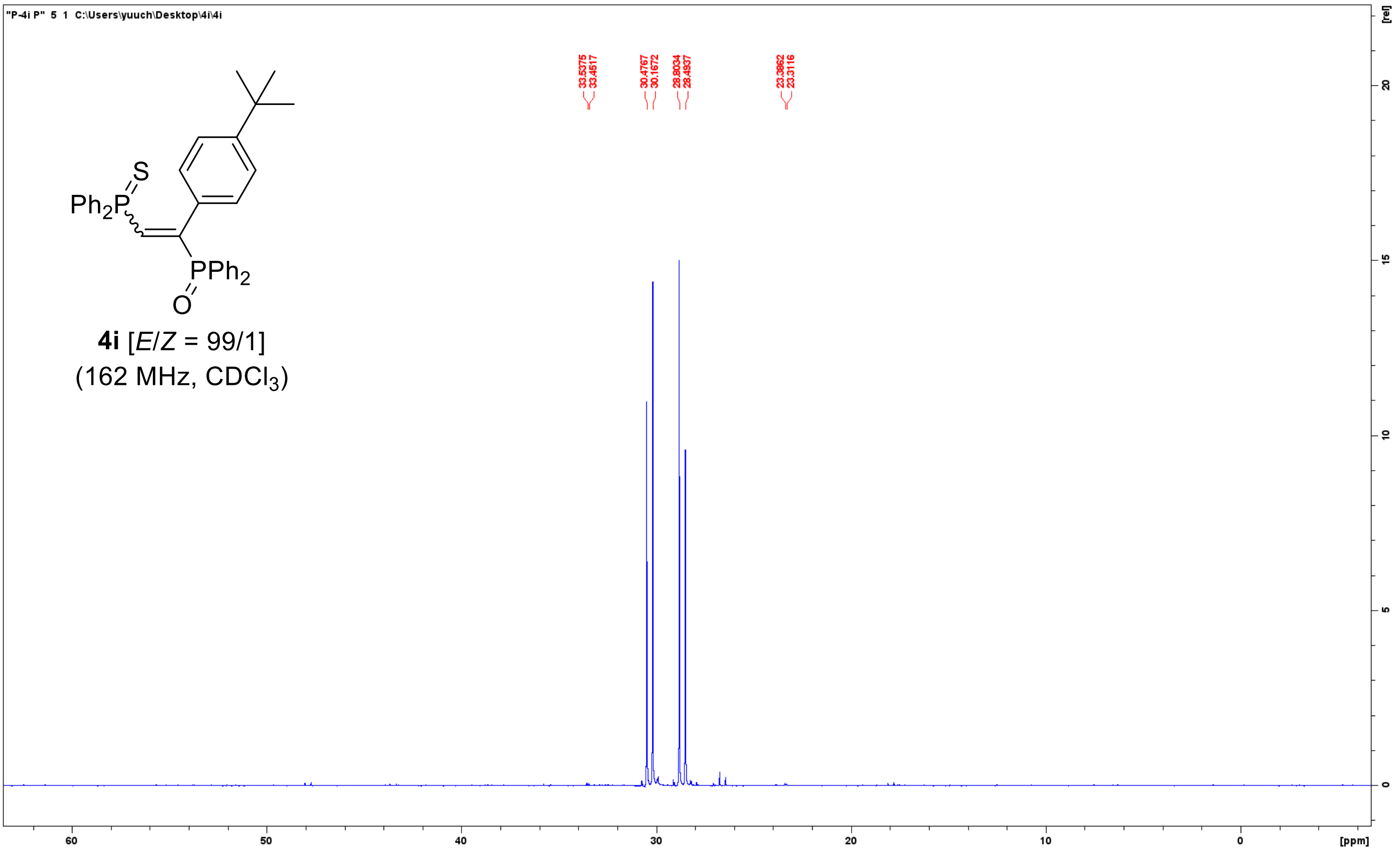

"P-4i P" 5 1 C:\Users\yuuch\Desktop\4i\4i
Ph2P
S
PPh2
O
4i [*E*/*Z* = 99/1]
(162 MHz, CDCl3)
33.5375
33.4517
30.4767
30.1672
28.8034
28.4937
23.3662
23.3116
[rel]
[ppm]

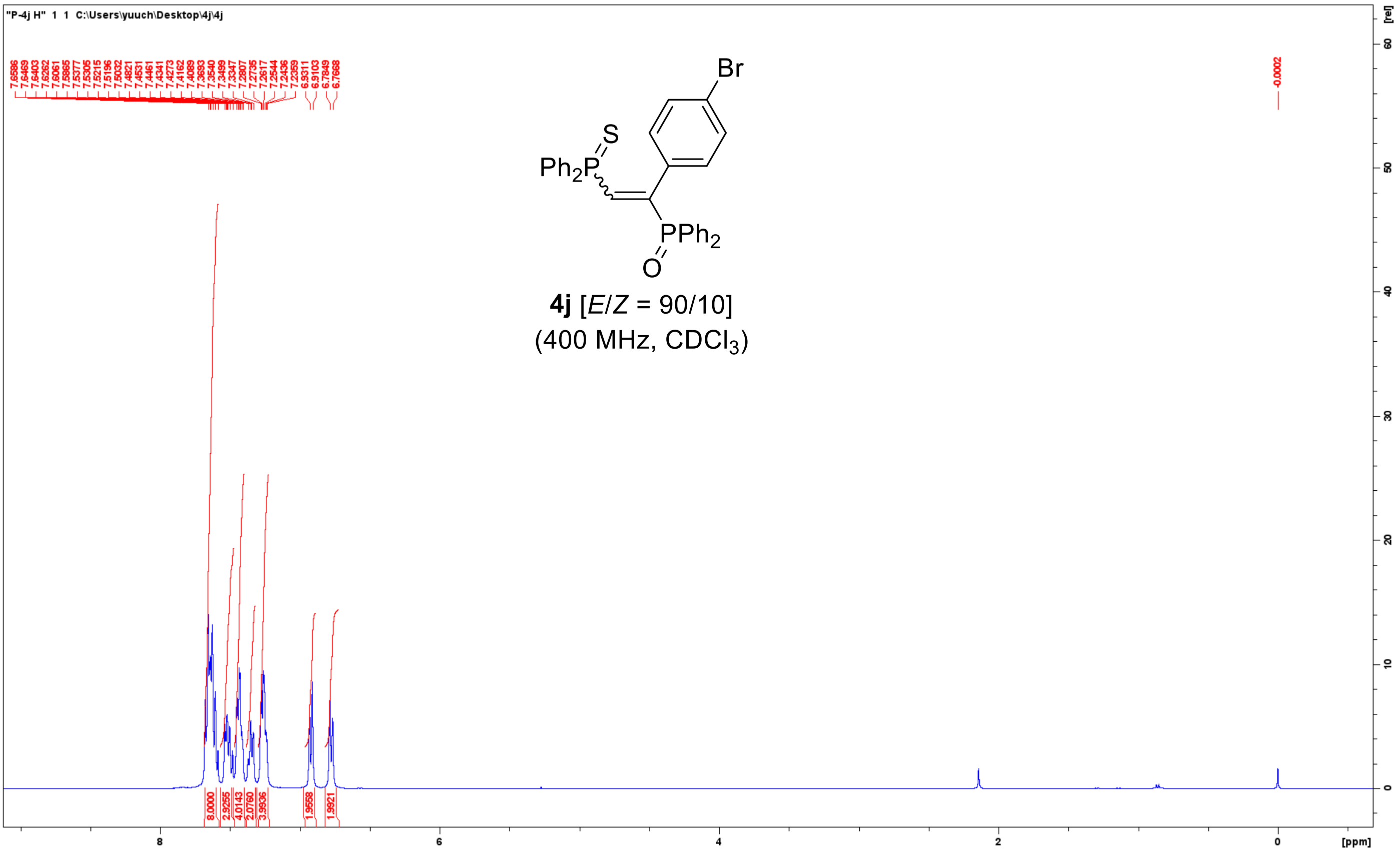
"P-4j H" 1 1 C:\Users\yuuch\Desktop\4j\4j
Br
S
Ph2P
PPh2
O
4j [E/Z = 90/10]
(400 MHz, CDCl3)
[ppm]
[rel]

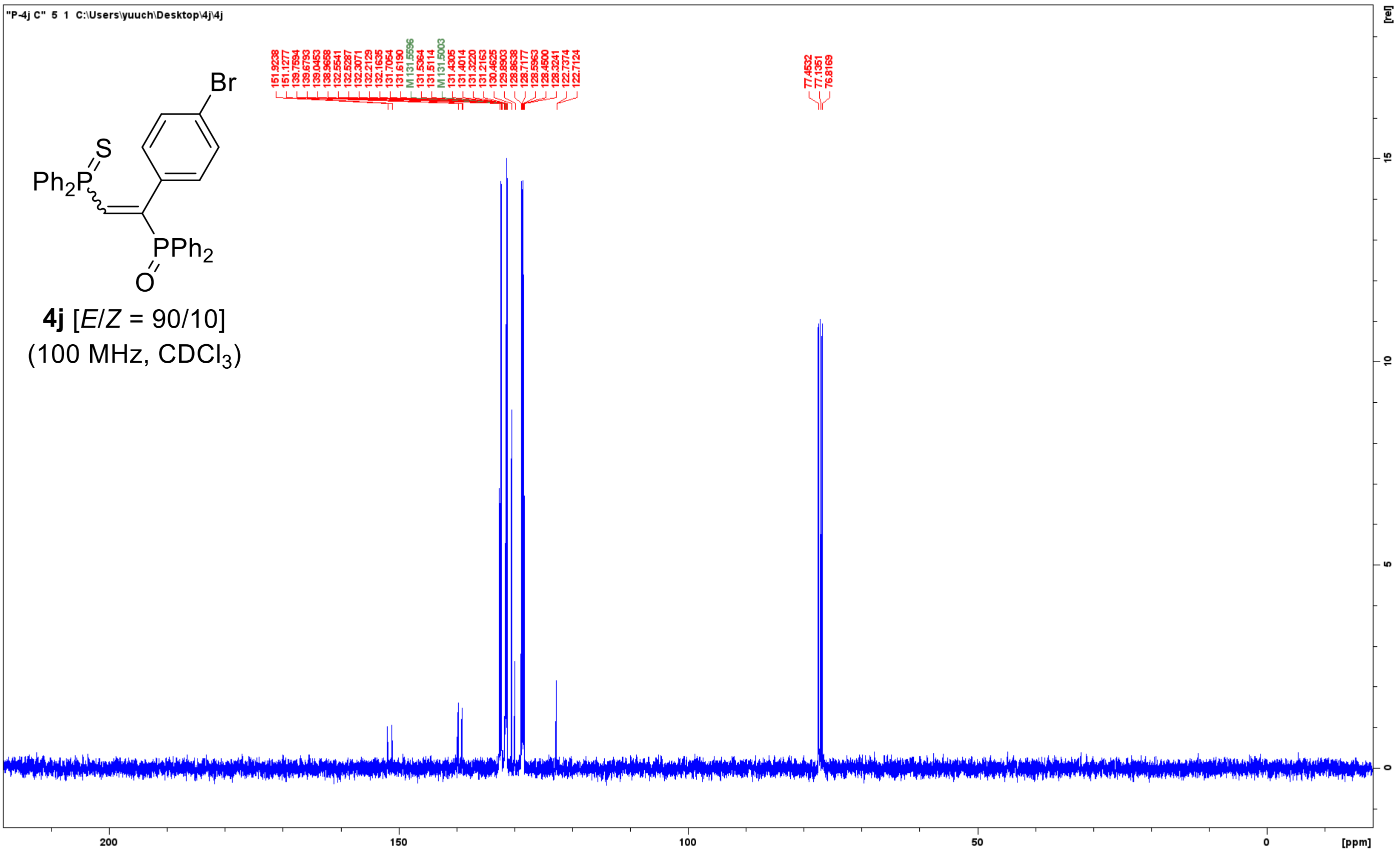
"P-4j C" 5 1 C:\Users\yuuch\Desktop\4j\4j
Br
S
Ph2P
PPh2
O
4j [E/Z = 90/10]
(100 MHz, CDCl3)
[ppm]
[rel]

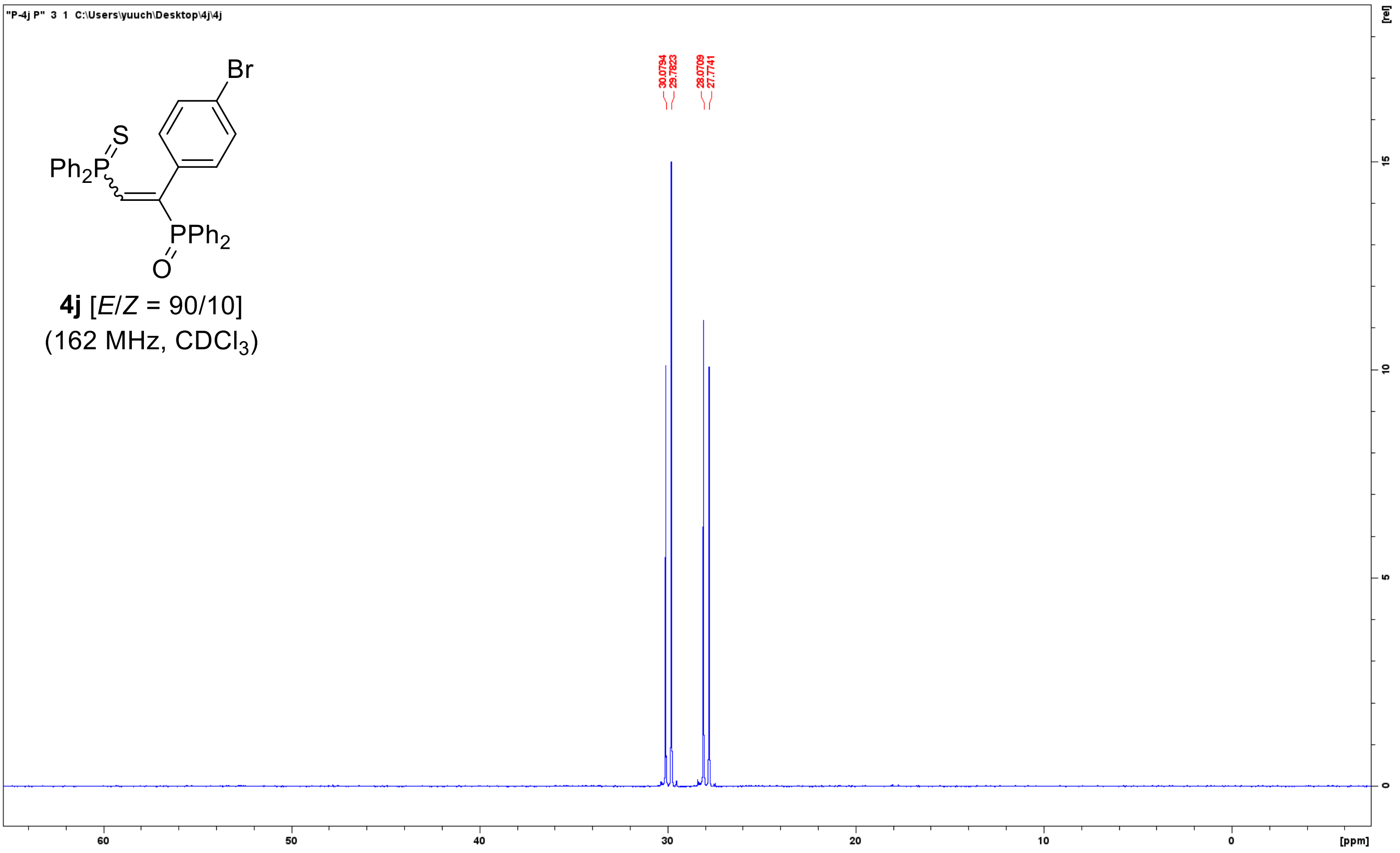
"P-4j P" 3 1 C:\Users\yuuch\Desktop\4j\4j
Br
S
Ph_2P
PPh_2
O
4j [*E*/*Z* = 90/10]
(162 MHz, $CDCl_3$)
30.0794
29.7823
28.0709
27.7741
[ppm]
[rel]

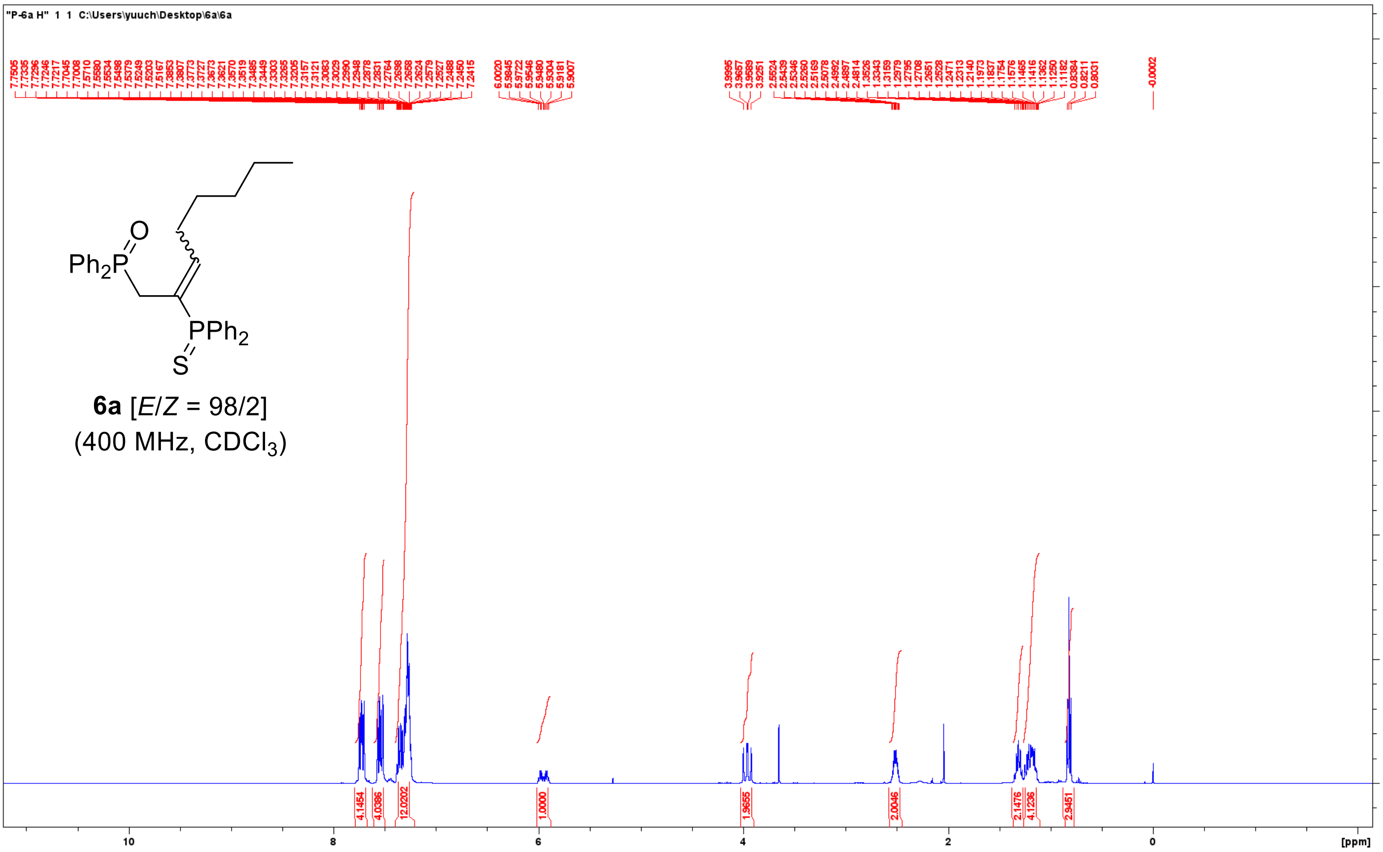
"P-6a H" 1 1 C:\Users\yuuch\Desktop\6a\6a
Ph2P=O
PPh2
S
6a [*E*/*Z* = 98/2]
(400 MHz, CDCl3)
[ppm]
[rel]

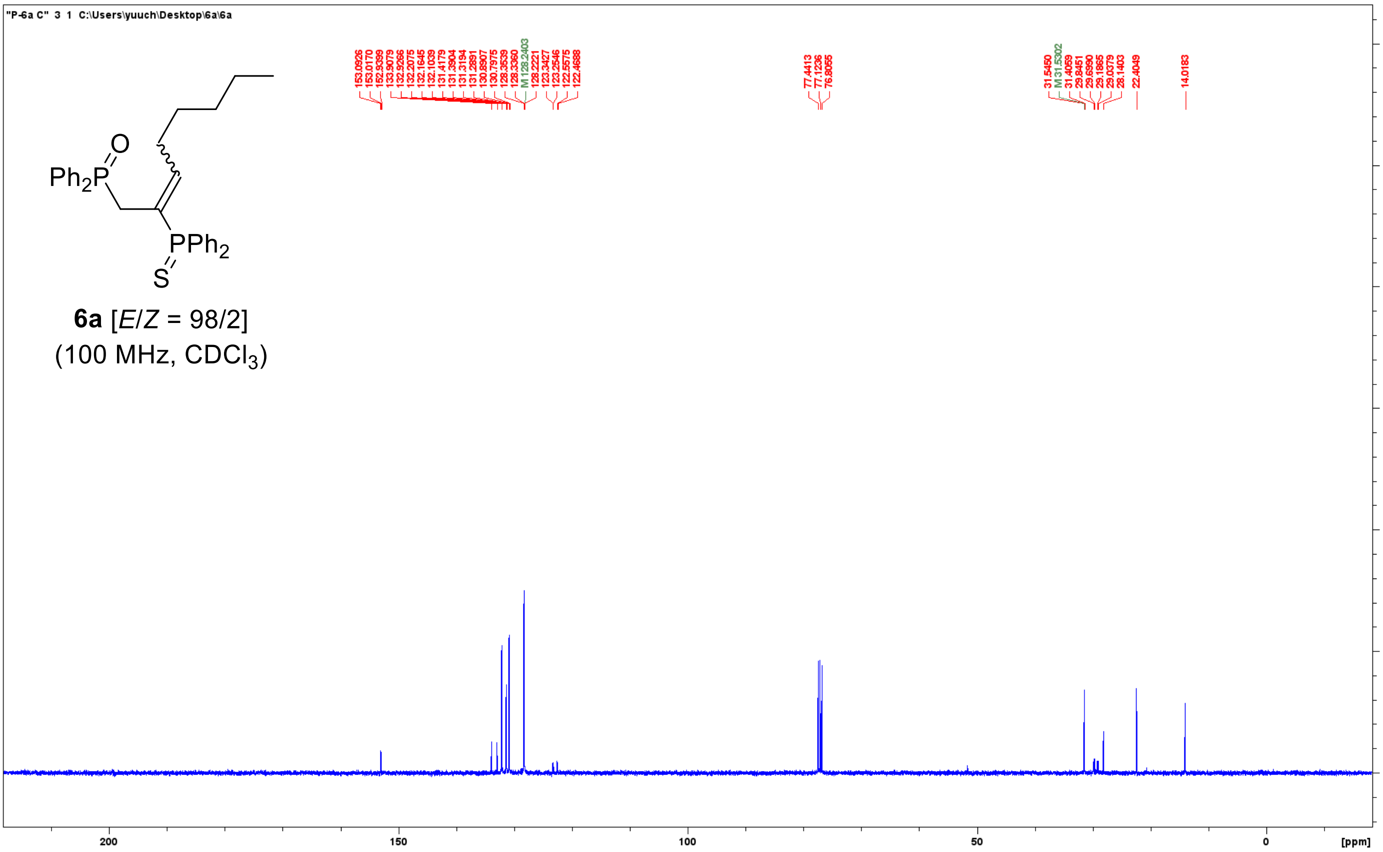
"P-6a C" 3 1 C:\Users\yuuch\Desktop\6a\6a
Ph2P=O
PPh2
S
6a [*E*/*Z* = 98/2]
(100 MHz, CDCl3)
[ppm]
[rel]

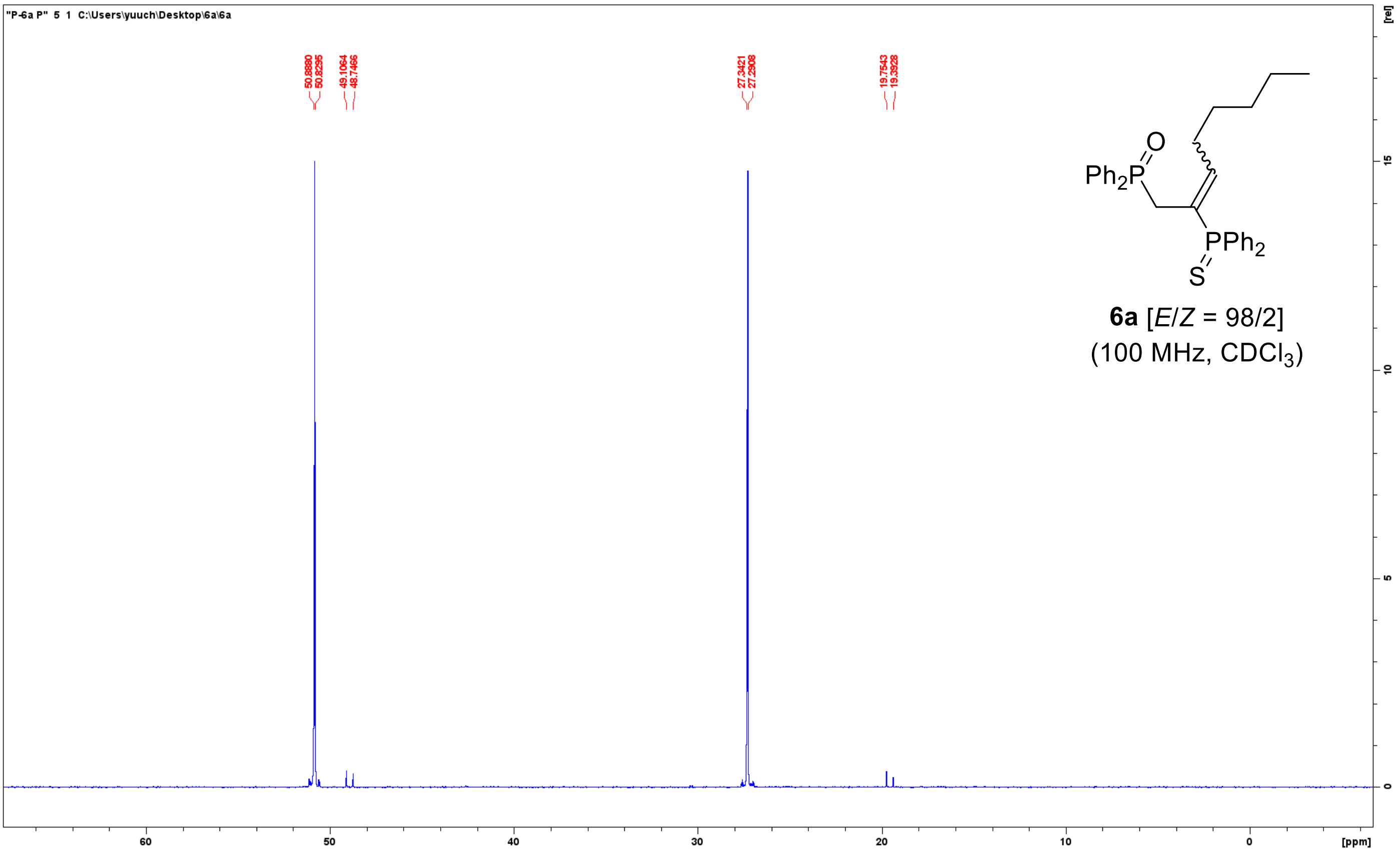
"P-6a P" 5 1 C:\Users\yuuch\Desktop\6a\6a
50.8880
50.8295
49.1064
48.7466
27.3421
27.2908
19.7543
19.3928
Ph2P
O
PPh2
S
6a [E/Z = 98/2]
(100 MHz, CDCl3)
60
50
40
30
20
10
0
[ppm]
[rel]
15
10
5
0

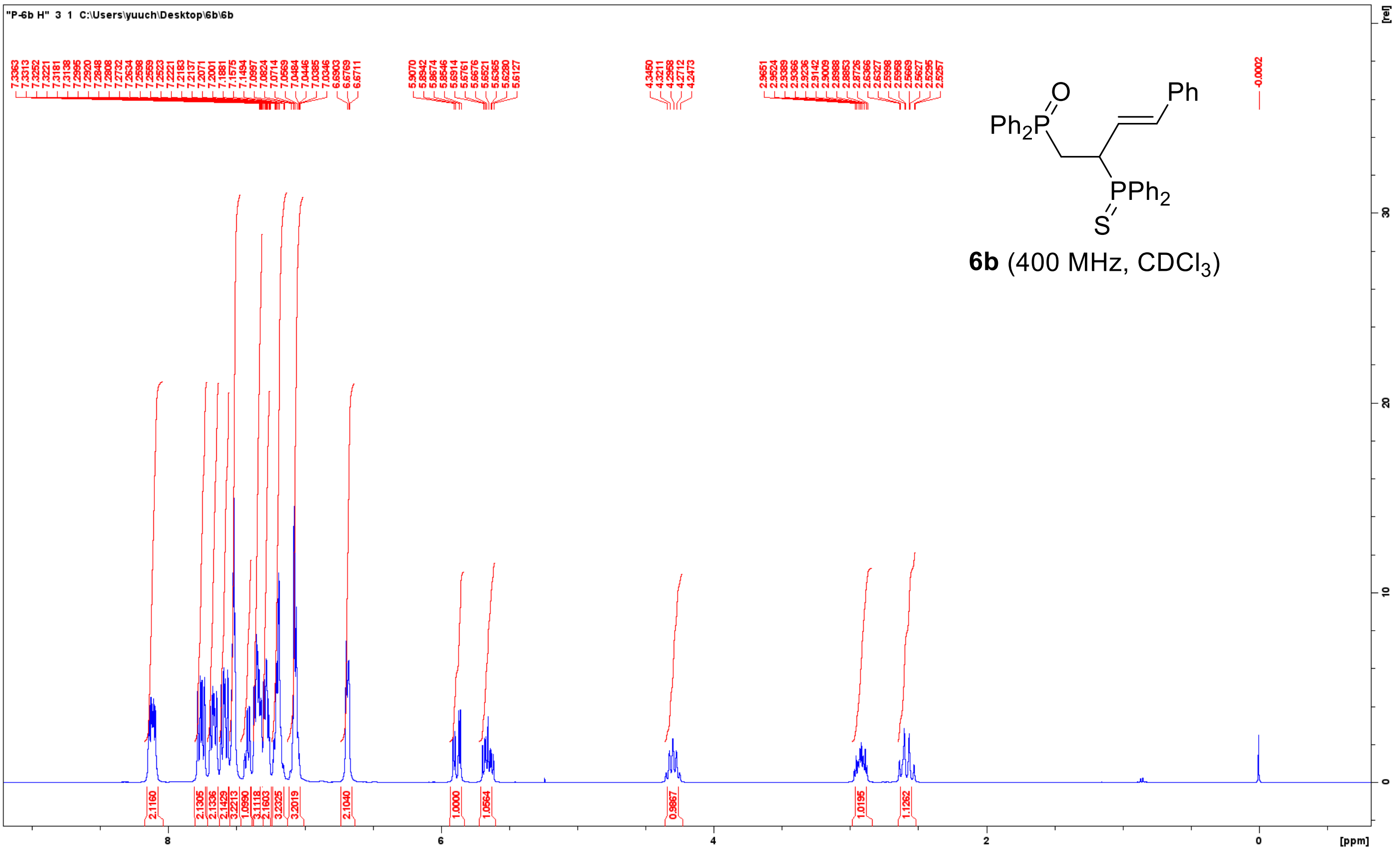
"P-6b H" 3 1 C:\Users\yuuch\Desktop\6b\6b
6b (400 MHz, CDCl3)
[ppm]
[rel]

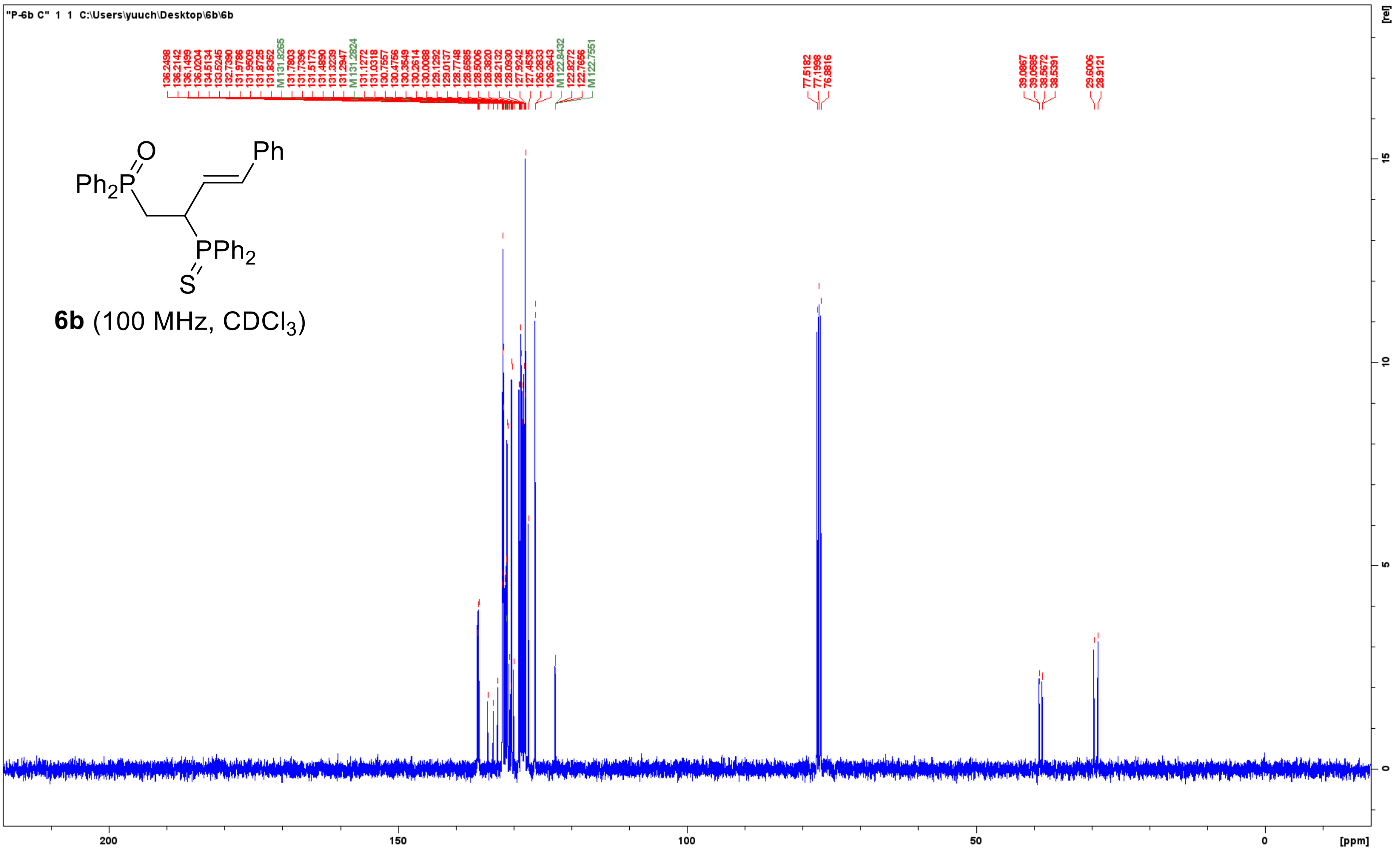
"P-6b C" 1 1 C:\Users\yuuch\Desktop\6b\6b
6b (100 MHz, CDCl3)
[ppm]
[rel]

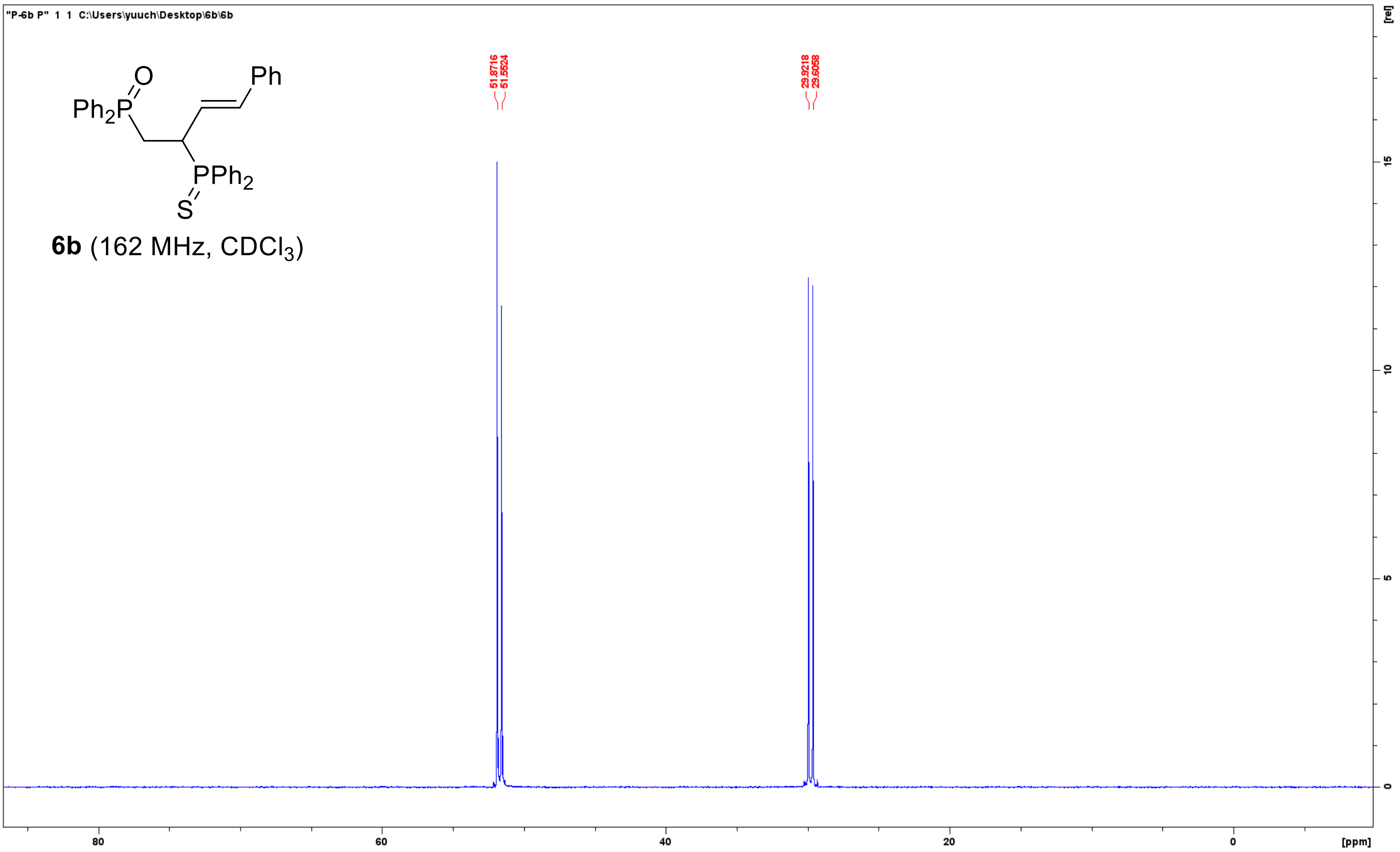
"P-6b P" 1 1 C:\Users\yuuch\Desktop\6b\6b
O
Ph
Ph2P
PPh2
S
6b (162 MHz, CDCl3)
51.8716
51.5524
29.9218
29.6058
[rel]
15
10
5
0
80
60
40
20
0
[ppm]

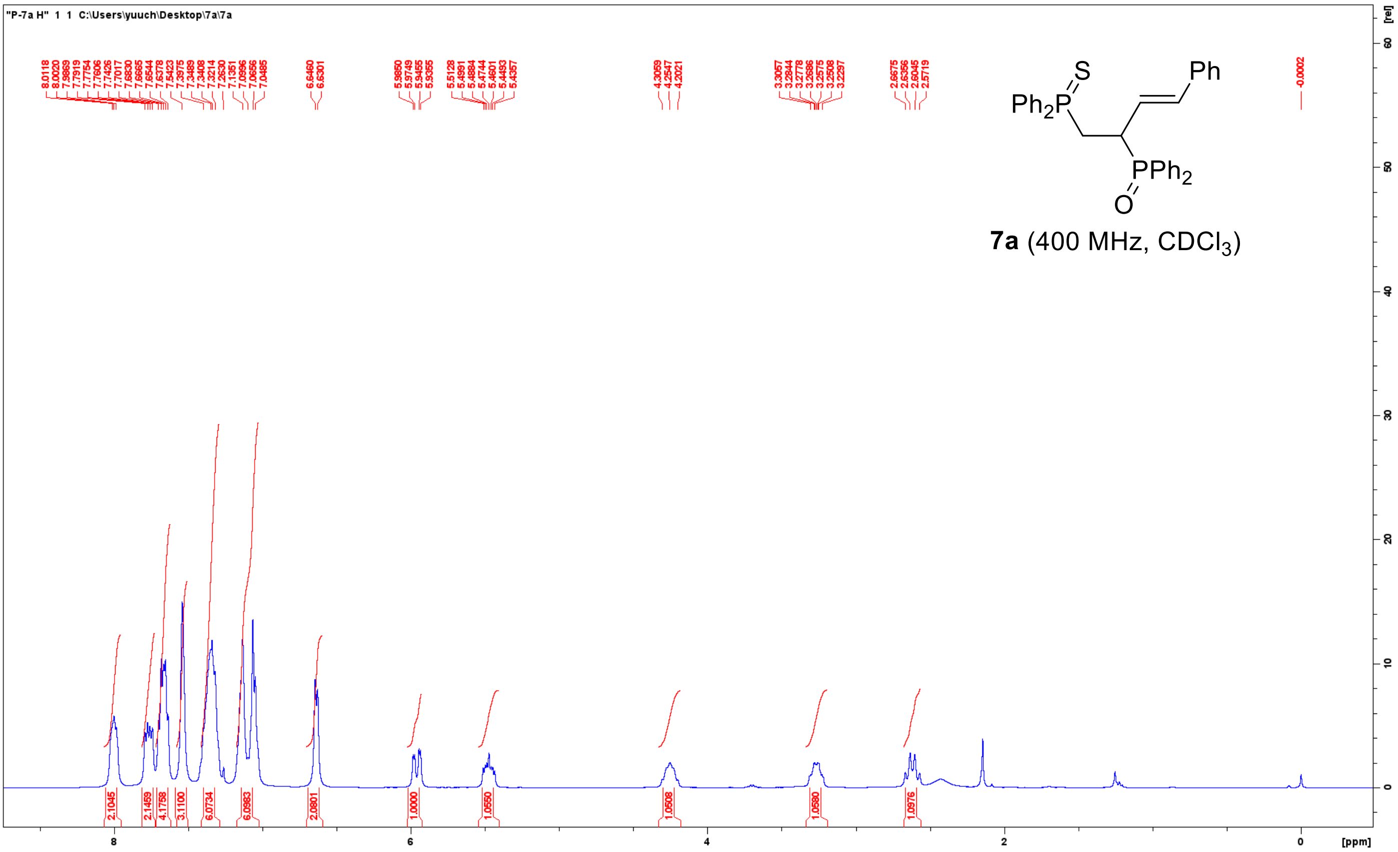
"P-7a H" 1 1 C:\Users\yuuch\Desktop\7a\7a
7a (400 MHz, CDCl3)
[ppm]
[rel]

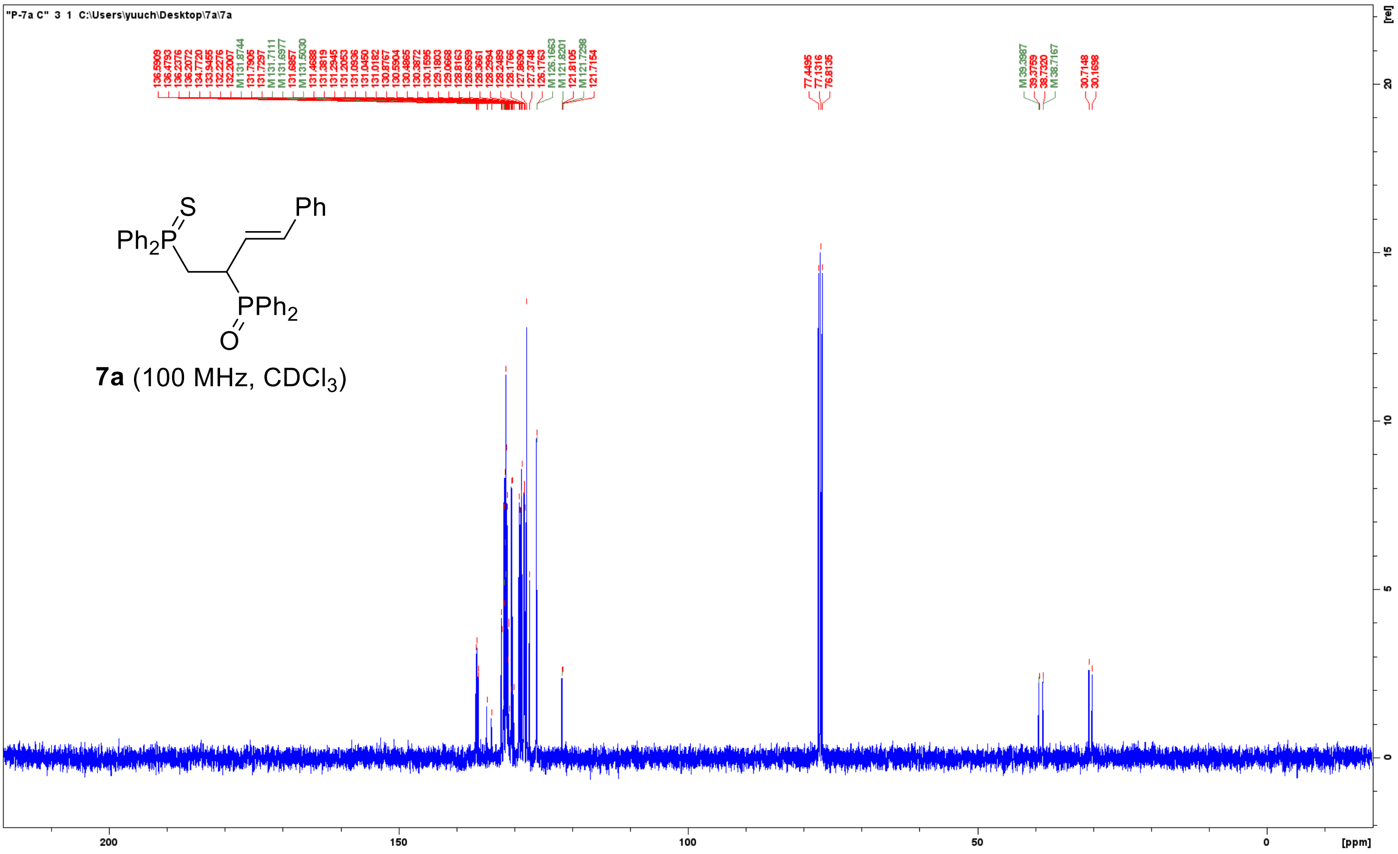
"P-7a C" 3 1 C:\Users\yuuch\Desktop\7a\7a
7a (100 MHz, CDCl3)
[ppm]
[rel]

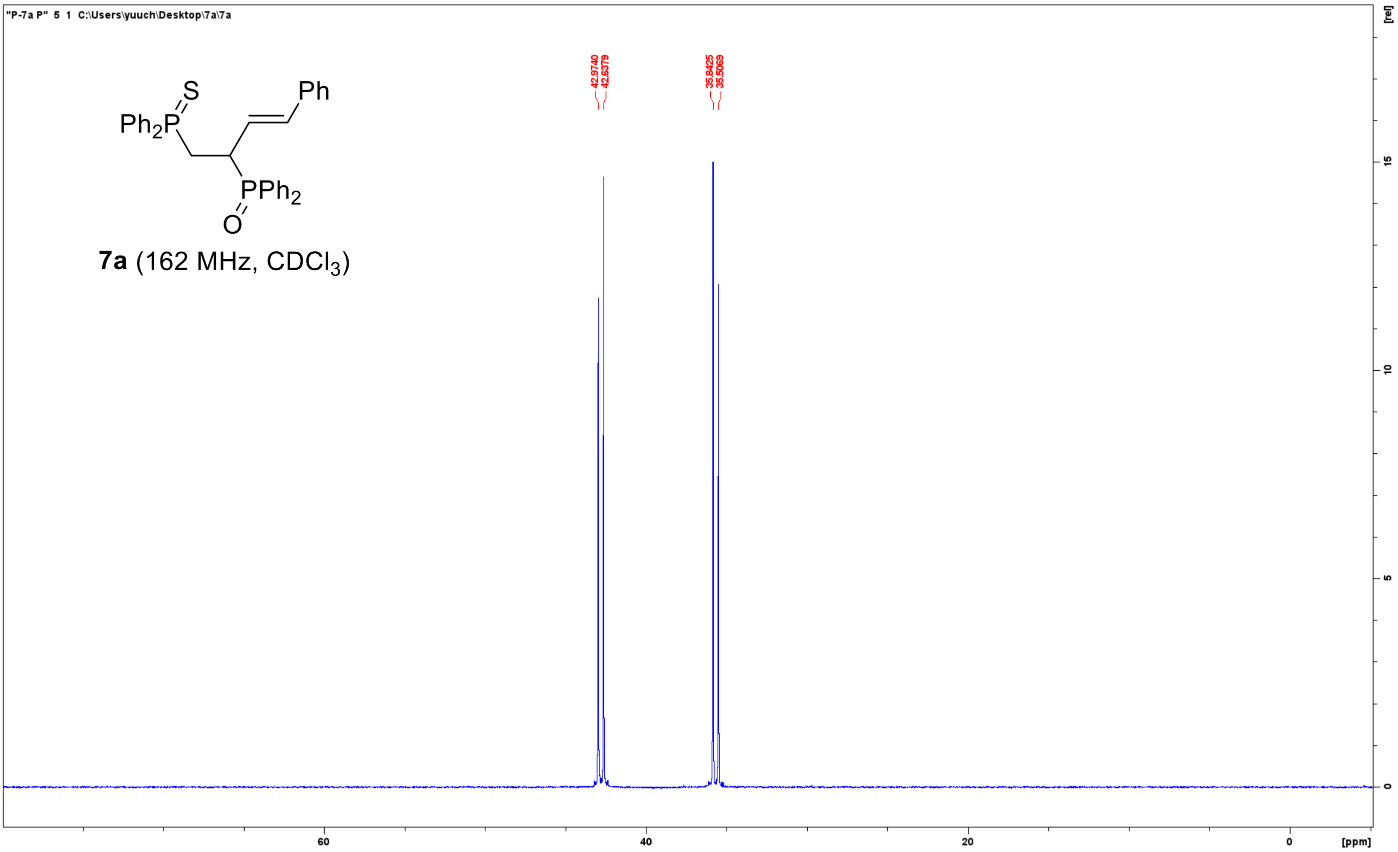
"P-7a P" 5 1 C:\Users\yuuch\Desktop\7a\7a
Ph
S
Ph2P
PPh2
O
7a (162 MHz, CDCl3)
42.9740
42.6379
35.8425
35.5069
[rel]
15
10
5
0
60
40
20
0
[ppm]
